# Cryo‐EM structure of the octameric pore of *Clostridium perfringens* β‐toxin

**DOI:** 10.15252/embr.202254856

**Published:** 2022-10-10

**Authors:** Julia Bruggisser, Ioan Iacovache, Samuel C Musson, Matteo T Degiacomi, Horst Posthaus, Benoît Zuber

**Affiliations:** ^1^ Institute of Animal Pathology, Vetsuisse‐Faculty University of Bern Bern Switzerland; ^2^ Institute of Anatomy, Medical Faculty University of Bern Bern Switzerland; ^3^ Department of Physics Durham University Durham UK

**Keywords:** beta toxin, *Clostridium perfringens*, cryo‐EM, hemolysin, pore‐forming toxin, Membranes & Trafficking, Microbiology, Virology & Host Pathogen Interaction, Structural Biology

## Abstract

*Clostridium perfringens* is one of the most widely distributed and successful pathogens producing an impressive arsenal of toxins. One of the most potent toxins produced is the *C. perfringens* β‐toxin (CPB). This toxin is the main virulence factor of type C strains. We describe the cryo‐electron microscopy (EM) structure of CPB oligomer. We show that CPB forms homo‐octameric pores like the hetero‐oligomeric pores of the bi‐component leukocidins, with important differences in the receptor binding region and the N‐terminal latch domain. Intriguingly, the octameric CPB pore complex contains a second 16‐stranded β‐barrel protrusion atop of the cap domain that is formed by the N‐termini of the eight protomers. We propose that CPB, together with the newly identified Epx toxins, is a member a new subclass of the hemolysin‐like family. In addition, we show that the β‐barrel protrusion domain can be modified without affecting the pore‐forming ability, thus making the pore particularly attractive for macromolecule sensing and nanotechnology. The cryo‐EM structure of the octameric pore of CPB will facilitate future developments in both nanotechnology and basic research.

## Graphical abstract

*Clostridium perfringens* β‐toxin (CPB) is a beta‐pore‐forming toxin of the hemolysin family and an essential virulence factor of type C strains causing fatal necrotic enteritis in animals and humans. This study reports the cryo‐electron microscopy structure of the oligomeric CPB‐pore which can facilitate future developments in both nanotechnology and basic research.

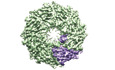

## Introduction

One of the most common and evolutionary conserved bacterial virulence mechanisms is the secretion of protein toxins that disrupt cellular membranes by pore formation. Such pore‐forming toxins (PFTs) are used by the pathogens to invade, survive, and disseminate in their hosts. Despite their large diversity, bacterial PFTs share common features. Most are secreted as water‐soluble monomers and bind to target cells via membrane receptors. Receptor binding leads to an increase in the local concentration, oligomerization, and insertion of a stable pore in the cell membrane. This allows uncontrolled exchanges between the extracellular and intracellular milieus, disturbs cellular homeostasis, and leads to diverse reactions ranging from defense mechanisms to cell death (Dal Peraro & van der Goot, 2016). Because of their nearly universal presence in bacterial pathogens, common structures and mechanisms used by PFTs are promising targets for novel anti‐virulence strategies as alternatives or supplementation to increasingly ineffective antibiotic treatments. Moreover, PFTs have gathered much interest in the scientific community beyond bacterial infections. The nano‐sized pores that they form are used for “sensing” biomolecules. Many nanopore applications are based on α‐hemolysin (Hla), the prototype hemolysin‐like β‐PFT secreted by *Staphylococcus aureus* (Kasianowicz *et al*, 1996) as well as aerolysin from *Aeromonas hydrophila* (Cao *et al*, 2019).

The human and animal pathogen *Clostridium perfringens* causes many severe diseases such as wound infections, septicemia, food poisoning, enterotoxemia, and enteritis (Songer, 1996, 2010; Kiu & Hall, 2018). The bacterium can produce a large arsenal of exotoxins, in particular PFTs that cross the membrane as a β‐barrel (β‐PFT). The largest group among them, with currently 11 known members, are the hemolysin‐like β‐PFTs (Popoff & Bouvet, 2009; Popoff, 2014; Mehdizadeh Gohari *et al*, 2015; Lacey *et al*, 2019). Despite their widespread use by the pathogen, their role in diseases is incompletely understood. One of the most potent toxins produced by *C. perfringens* is the β‐toxin (CPB; Uzal *et al*, 2014). CPB is secreted by *C. perfringens* type C strains and is essential in the pathogenesis of a lethal necrotic enteritis in many animal species and humans (Uzal *et al*, 2014). CPB contributes to the intestinal damages by targeting endothelial cells and potentially thrombocytes, leading to vascular damage and hemorrhage (Posthaus *et al*, 2020). Based on sequence homology between mature CPB and other bacterial toxins (Appendix Fig S1 and S2), the toxin is a member of the hemolysin‐like family of β‐PFTs. Within this group, CPB is most closely related to *C. perfringens* δ‐toxin (46% identity) and NetB (39% identity). In addition, it shows a 41% identity with the newly discovered enterococcal EPX4 toxin (Xiong *et al*, 2022). It shares lower sequence homology to the staphylococcal toxins Hla (26% identity) and the bi‐component leukocidins (Notredame *et al*, 2000; Robert & Gouet, 2014). Recently, we determined the molecular basis for the specificity of CPB toward endothelial and leukocytic cells by showing that PECAM‐1/CD31, an endothelial and leukocytic adhesion molecule, serves as its cellular receptor (Bruggisser *et al*, 2020; Tarek *et al*, 2021).

So far however, no structural information has been available for CPB. In the present study, we describe the cryo‐electron microscopy (EM) structure of CPB in SMA discs, which likely represents the membrane‐inserted pore form, at near atomic resolution. We show that CPB forms a homo‐oligomeric pore with a novel N‐terminal β‐barrel replacing the typical hemolysin latch domain. We propose that the N‐terminal β‐hairpin stabilizes the monomeric protein in solution and influences the pore conductivity.

Our results have important implications for comparative studies on related toxins and the rational design of novel anti‐virulence strategies against clostridial diseases. In addition, the unique features of the N‐terminal β‐barrel make CPB an attractive candidate for applications in nanotechnology.

## Results and Discussion

### Formation of the CPB oligomeric pore

**Figure 1:**
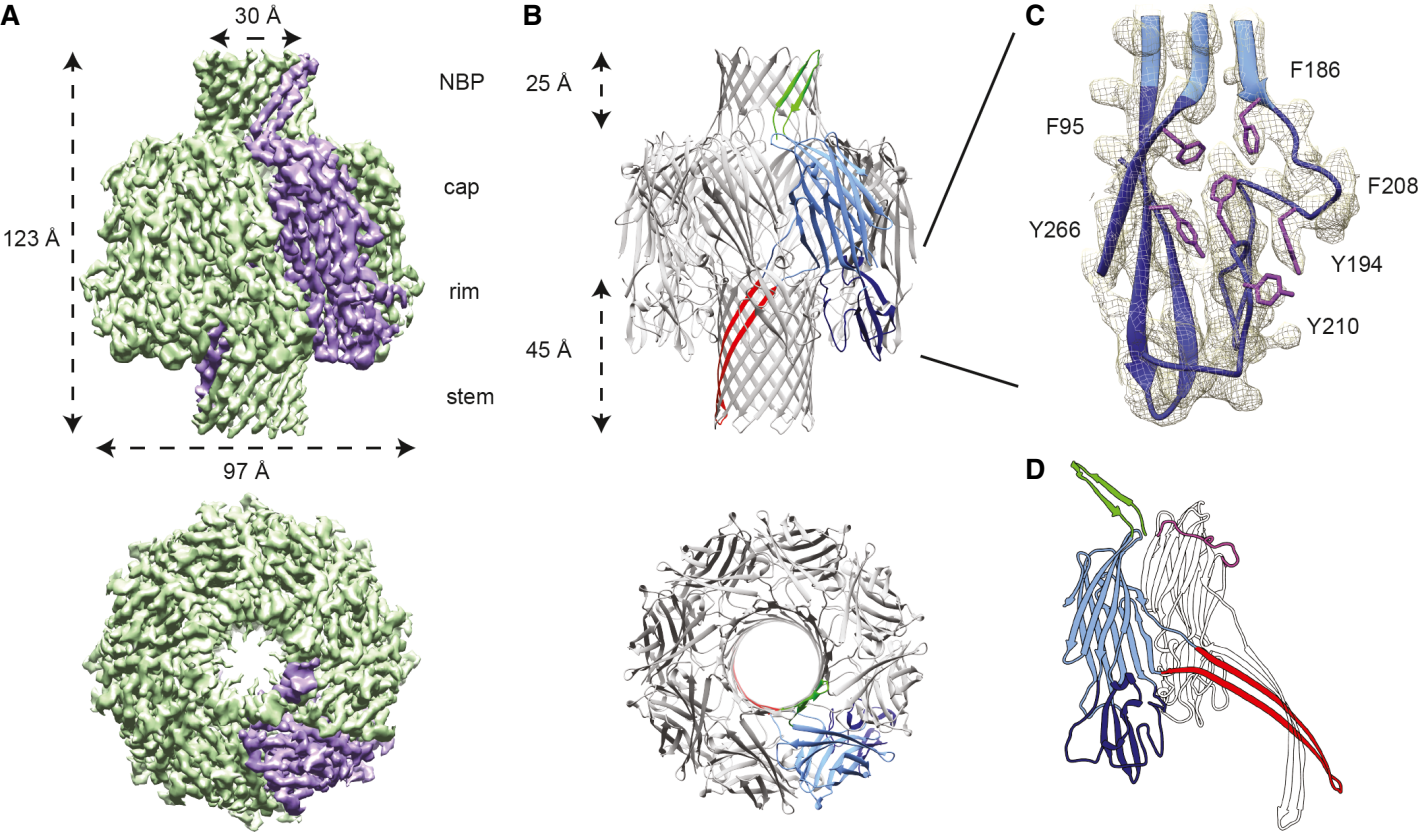
Structure of the CPB pore Side and top views of the 3.8 Å sharpened cryo‐EM map of the *Clostridium perfringens* β‐toxin (CPB) with one protomer highlighted in purple. The map shows an extended 123 Å particle with protrusions on both sides. The diameter of the particles is 97 Å with a visible channel of ~ 30 Å diameter.Model of the CPB oligomer showing a homo‐octameric pore in top view and side view. The dimensions of the two β‐barrels are indicated as measured from Cα to Cα. One protomer is colored by domains: green—N‐terminal β‐barrel protrusion; light blue—β‐sandwich cap domain; dark blue—rim domain; red—stem domain.Magnified view of the aromatic pocket of the rim domain docked in the cryo‐EM map showing the density of the aromatic side chains. Residues are indicated in proximity to their respective densities.Cartoon model of the CPB protomer in the oligomer color coded as in (B) compared to a protomer extracted from the Hla oligomer. The N‐terminus of Hla is highlighted in purple.

**Figure EV1:**
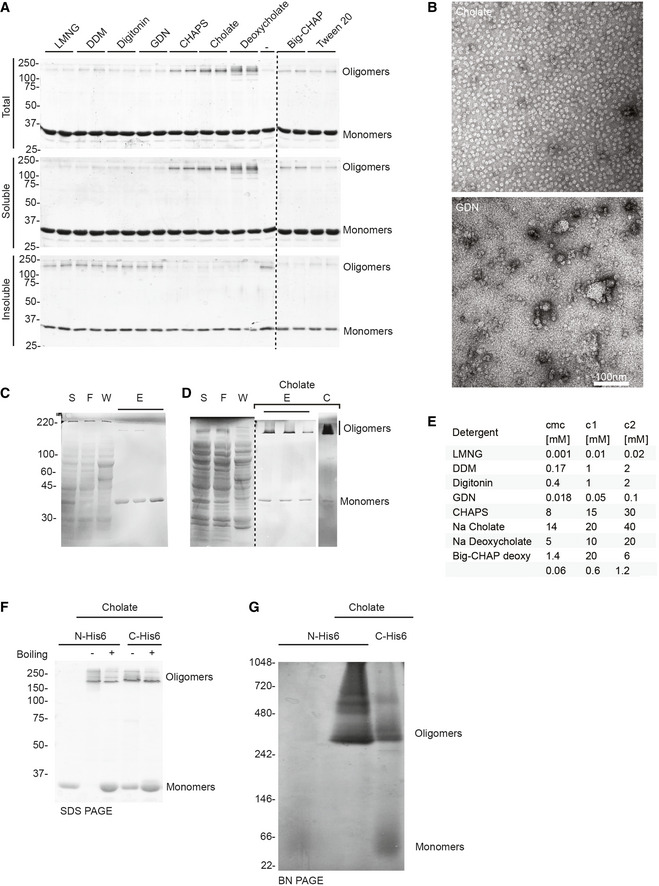
Oligomerization and solubility of CPB oligomers in different detergents ADetergent screen showing cholate and deoxycholate as the best candidates for purification and solubilization of *Clostridium perfringens* β‐toxin (CPB) pores. Each detergent was added to the CPB sample for 16 h at 4° and then the sample was centrifuged at 15,000 *g* for 10 min. The pelleted insoluble fraction was recovered in sample buffer and loaded on sodium dodecyl sulphate polyacrylamide gel electrophoresis (SDS‐PAGE) together with the soluble fraction and the initial (total) fractions.BNegative stain of representative CPB oligomers after purification in 30 mM cholate with evenly spread oligomers (top) vs after exchanging cholate with 0.1 mM GDN with aggregates and background particles (bottom).C, DSDS‐PAGE gels of His6‐CPB purification in the absence (C) and presence (D) of cholate. 10 μl of supernatant (S), flow through (F), and elution (E) fractions were loaded, and gels stained with Coomassie stain. Most oligomers precipitate in the absence of detergent during the purification, whereas after concentration (C) almost all CPB shifted into the oligomeric state in the presence of cholate.ETable of the detergents used for the screen, their CMC and their concentration.F, GCharacterization of N‐ and C‐terminal tagged CPB constructs by gel electrophoretic analysis under denaturing (F) and native (G) conditions. SDS‐PAGE gel analysis of CPB samples (1 μg per lane) showing monomeric CPB in the absence of detergent and SDS‐resistant oligomers in the presence of cholate. CPB samples containing cholate were boiled for 5 min at 95°C (+) or not (−).

**Figure EV2:**
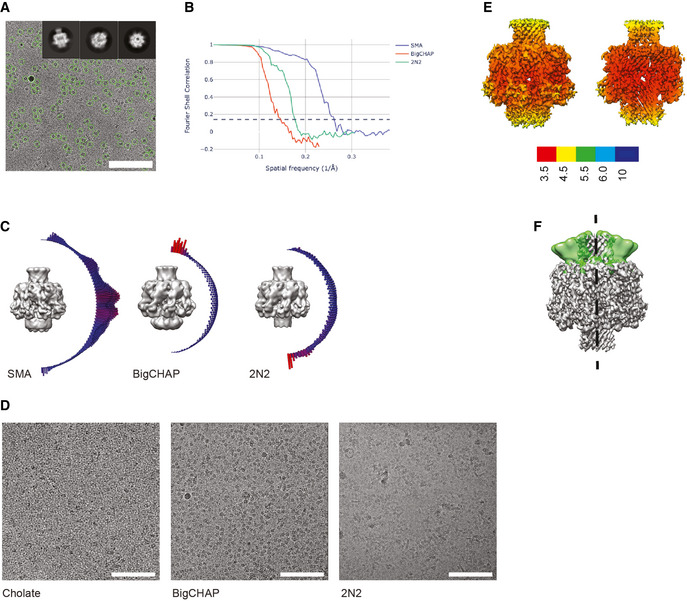
Cryo‐EM of CPB Electron micrograph of a typical field of view of oligomeric *Clostridium perfringens* β‐toxin (CPB). Automatically picked particles shown in green. Inset showing characteristic 2D class averages, side, tilted, top. Scale bar is 100 nm.FSC showing the resolution of CPB oligomers in different reconstruction conditions with SMA giving the best orientation of the sample on the grid.Refined cryo‐EM maps and angular distribution of particles of CPB reconstituted in SMA, BigCHAP or 2N2 nanodiscs.Representative micrographs of the data in panels B and C.Local resolution estimate performed in RELION showing the cap and rim domains at the highest resolution.Position of the NBP at the extremes of the multibody analysis performed in RELION (in green) compared to the CPB map (gray).

To investigate the pore formed by CPB by single particle cryo‐EM, we screened for a suitable detergent for oligomerization. CPB spontaneously assembles into SDS‐resistant oligomeric species when stored in solution leading to partial precipitation of the protein. Purification in the presence of cholate followed by detergent removal and exchange with 2.5% SMA (Knowles *et al*, 2009) led to a homogeneous distribution of pores suitable for single particle cryo‐EM (Fig EV1A–G). Interestingly, the synthetic nanodiscs did not require addition of lipids suggesting that the SMA is able to directly wrap around the β‐barrel of the CPB pore keeping the complex soluble. Particle distribution, orientation, and sample quality in SMA were better than either in detergent or inserted in protein‐based nanodiscs (Fig EV2A–D). The average diameter of the particles was ~100 Å. Two‐dimensional classification of the particles revealed CPB pores with eight‐fold symmetry. Further refinement and postprocessing resulted in an electron density map at an estimated 3.8 Å resolution (Figs 1A and C, and EV2E and F). This allowed us to unambiguously build a model for the CPB octameric pore except for two loops in the rim domain (Glu76–Ser89, Ala283–Pro287), 4N‐terminal amino acids and several amino acids with unresolved side chains (Fig 1B).

### Molecular architecture of the CPB pore

**Figure EV3:**
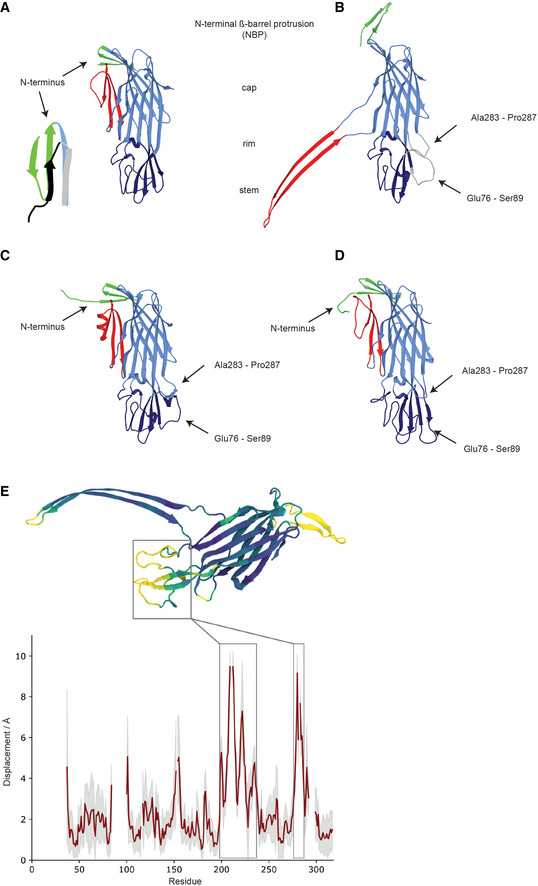
Modeling the missing features of the CPB model and structural comparison of CPB and γ‐hemolysin AModel of *Clostridium perfringens* δ‐toxin (2YGT) using the same color code as in Fig 1 showing the position of its N‐terminus folded back as an additional strand with the inset showing a comparison between the N‐terminus of the δ‐toxin (green) and the N‐terminus of *Hla* (black—4YHD).BModel of a protomer of CPB extracted from the oligomer structure color coded as before. The missing loops in the rim domain are modeled and shown in gray. The missing loops are only shown as visual guide for the number of missing amino acids in the model as the map quality in those regions is not good enough for model building.C, DPrediction of the CPB monomer structure by AlphaFold (C) and RosettaFold (D) color coded as before. The predicted N‐terminus folds as the N‐terminus of δ‐toxin. The missing loops in the rim domains fold similarly to our modeling shown in (B) in the case of alphafold algorithm while RosettaFold modeling extends the longer missing loop into a long β‐sheet (D).EMonomers extracted from the *C. perfringens* β‐toxin (CPB) and γ‐hemolysin (PDB: 3B07) octamers were aligned (STAMP structural alignment), and the Euclidean distance of their paired Cα atoms measured. In the graph, the black line reports on the distances measured when using our CPB structure. The red line reports on the mean difference when using all structures extracted from our MD simulations for comparison, and the gray shaded region reports on the standard deviation. The protein rendering is colored according to the mean difference of simulated structures.

The cryo‐EM analysis of CPB clearly showed that like other members of the hemolysin‐like family, the CPB protomer is composed of a cap, a rim, and a stem domain (Johnstone *et al*, 2021). In addition to the prototypical features of the family members, CPB possesses a second β‐barrel on top of the cap domain. While the N‐terminus of Hla is located inside the cap domain and wraps the vestibule‐exposed surface of the adjacent protomer, the CPB N‐terminus protrudes from the cap as a short hairpin, which assembles to form a 16‐strand β‐barrel (Fig 1B and D). We termed it the N‐terminal β‐barrel protrusion (NBP). The cap domain consists of a β‐sandwich composed of two β‐sheets and short α‐helices. Two strands extend into the lower part of the molecule making up the rim domain. While the cap domain is one of the most conserved features within the hemolysin‐like family, interesting differences are found in the rim domain. Unfolded loops in this domain have been shown to bind the toxin receptor in LukGH leukocidin (CD11b) and phosphatidyl choline in both Hla and γ‐hemolysin (Trstenjak *et al*, 2020; Olson *et al*, 1999; Galdiero & Gouaux, 2004; Monma *et al*, 2004). Interestingly at position 210, CPB is the only family member to have a bulky aromatic residue, a tyrosine, pointing inside a pocket (Fig 1C). This pocket was shown to bind phosphocholine in Hla (Galdiero & Gouaux, 2004). Furthermore, CPB lacks a four‐residue stretch which lines the pocket. Among them a tryptophan which is important for phosphocholine binding in staphylococcal hemolysins (Monma *et al*, 2004). These differences are compatible with the fact that CPB main receptor is a membrane protein, whereas for both α‐ and γ‐hemolysins the main receptor is phosphatidylcholine (Dal Peraro & van der Goot, 2016; Bruggisser *et al*, 2020). The loops at the base of the rim domain were well resolved except for two stretches of 14 and 5 residues, respectively (Glu76–Ser89; Ala283–Pro287). Importantly, these stretches are predicted to be surface exposed (Fig EV3A–D). (Trstenjak *et al*, 2020). We compared the protomer structure in CPB and γ‐hemolysin octamers, respectively (Fig EV3E). The rim domain of the two proteins nearly perfectly overlaps except in the membrane‐proximal loop region, suggesting that the loops have different functions between the two toxins. This may indicate that this region of CPB could potentially bind its receptor. Indeed, a pairwise flexible alignment of CPB with other hemolysins shows that apart from the NBP the main structural differences are located in the rim domain, in particular in the flexible loops of the rim (Appendix Fig S3). This issue is out of the scope of this paper and will be investigated in subsequent studies. The stem domain is similar to the other family members. It contains a long, curved amphipathic hairpin, which is connected to the cap β‐sandwich through two short coils forming the transmembrane β‐barrel upon oligomerization. Overall, the CPB octamer forms a 123 Å‐long ring‐like structure with a widest outer diameter of 97 Å (Fig 1A and B). The channel runs along the 8‐fold symmetry axis. It goes through the 25‐Å long N‐terminal ß‐barrel, the central large 72,900 Å^3^ vestibule, formed by the cap domains, and the transmembrane β‐barrel.

### N‐terminal β‐barrel protrusion

**Figure 2:**
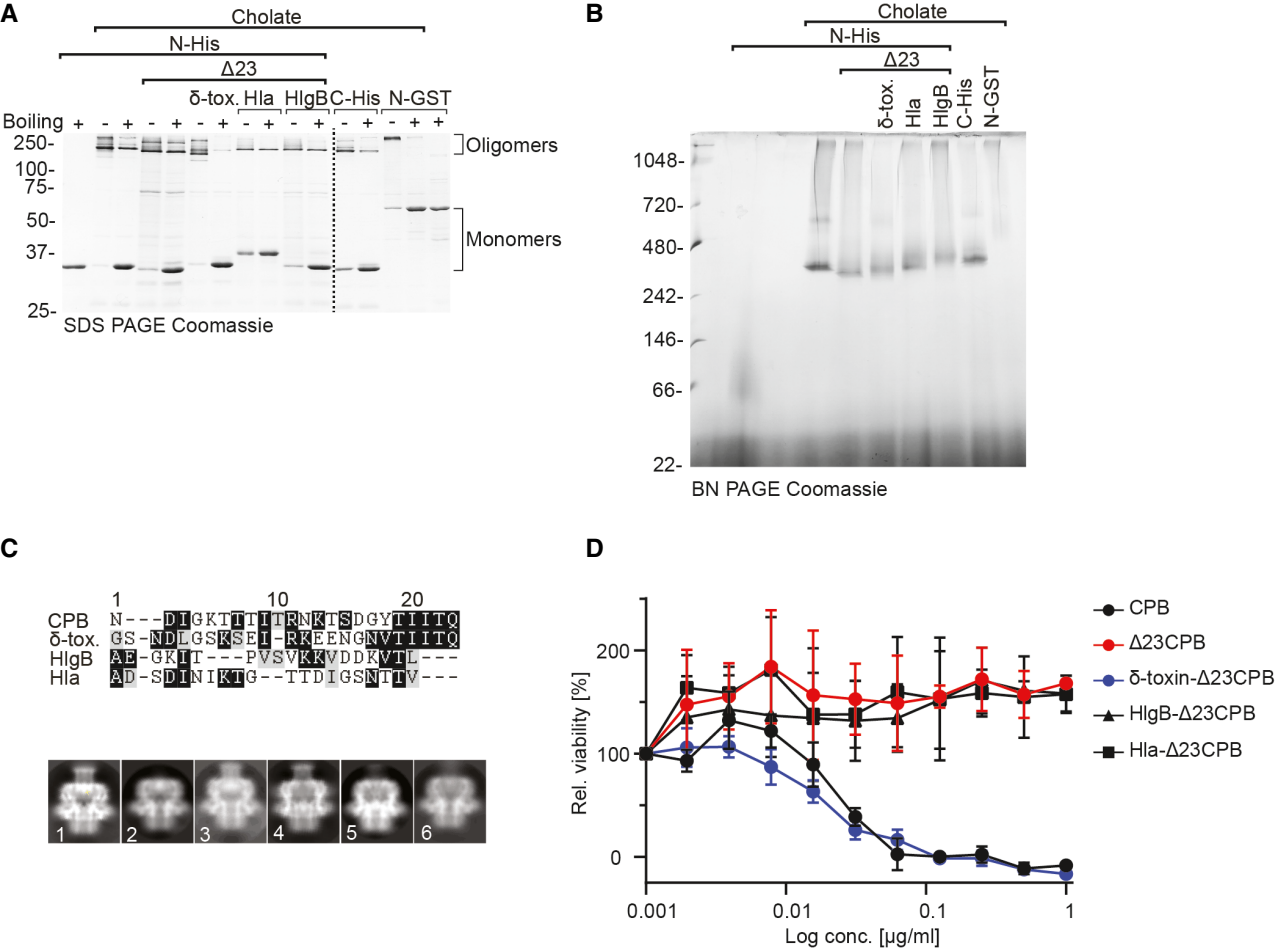
Investigating the N‐terminal domain of different hemolysins Characterization of *Clostridium perfringens* β‐toxin (CPB) constructs with different N‐termini by gel electrophoretic analysis under denaturing (A) and native (B) conditions. Sodium dodecyl sulphate polyacrylamide gel electrophoresis (SDS‐PAGE) gel analysis of CPB samples in cholate (1 μg per lane). CPB samples containing cholate were boiled for 5 min at 95°C (+) or not (−).Coomassie stained Blue native PAGE gel (4–16%) of CPB samples (10 μg per lane) showing oligomer formation for all different N‐termini constructs.Alignment of CPB N‐terminus with N‐termini of different hemolysins and cryo‐EM 2D classification and average of N‐terminally tagged WT CPB (1), Δ23CPB (2), δ‐toxin N‐terminus Δ23CPB (3), C‐terminally tagged WT CPB (4), Hla N‐terminus Δ23CPB (5), and HlgB N‐terminus Δ23CPB (6).Viability of HEK 293FT/CD31‐GFP cells (transduced with CD31‐GFP) as a percentage of untreated control cells after incubation with indicated concentrations of toxins (24 h, 37°C). Data (technical replicates) are represented as means (*n* = 4) ± SD.

To further investigate the NBP and its role, we constructed CPB mutants where the NBP was either deleted (Δ23CPB) or replaced by the equivalent N‐termini of *S. aureus* Hla (Hla‐Δ23CPB), γ‐hemolysin component B (HlgB‐Δ23CPB), or *C. perfringens* δ‐toxin (δ‐toxin‐Δ23CPB). While the modification of the N‐terminus seemed to affect protein solubility in *Escherichia coli*, with a much lower soluble protein yield for the chimeras when compared to the WT‐CPB, the cholate purified proteins still retained their ability to oligomerize and form pores (Fig 2A and B). 2D class averages of the different mutants showed the lack of the NBP density for the Δ23CPB mutant as well as a lack of a structured N‐terminus for the Hla‐Δ23CPB and HlgB‐Δ23CPB. The N‐terminus from δ‐toxin in the δ‐toxin‐Δ23CPB chimera seems to form an NBP similar to the WT‐CPB, suggesting that the N‐terminus of δ‐toxin oligomer might also adopt a β‐barrel conformation in its oligomeric form (Figs 2C and EV3A). Interestingly, the ability to form the NBP correlates with the cytotoxic activity. The truncated Δ23CPB, HlgB‐Δ23CPB, and Hla‐Δ23CPB were inactive, whereas activity was fully restored for the δ‐toxin‐Δ23CPB (Fig 2D).

### Channel properties and dynamics

**Figure 3:**
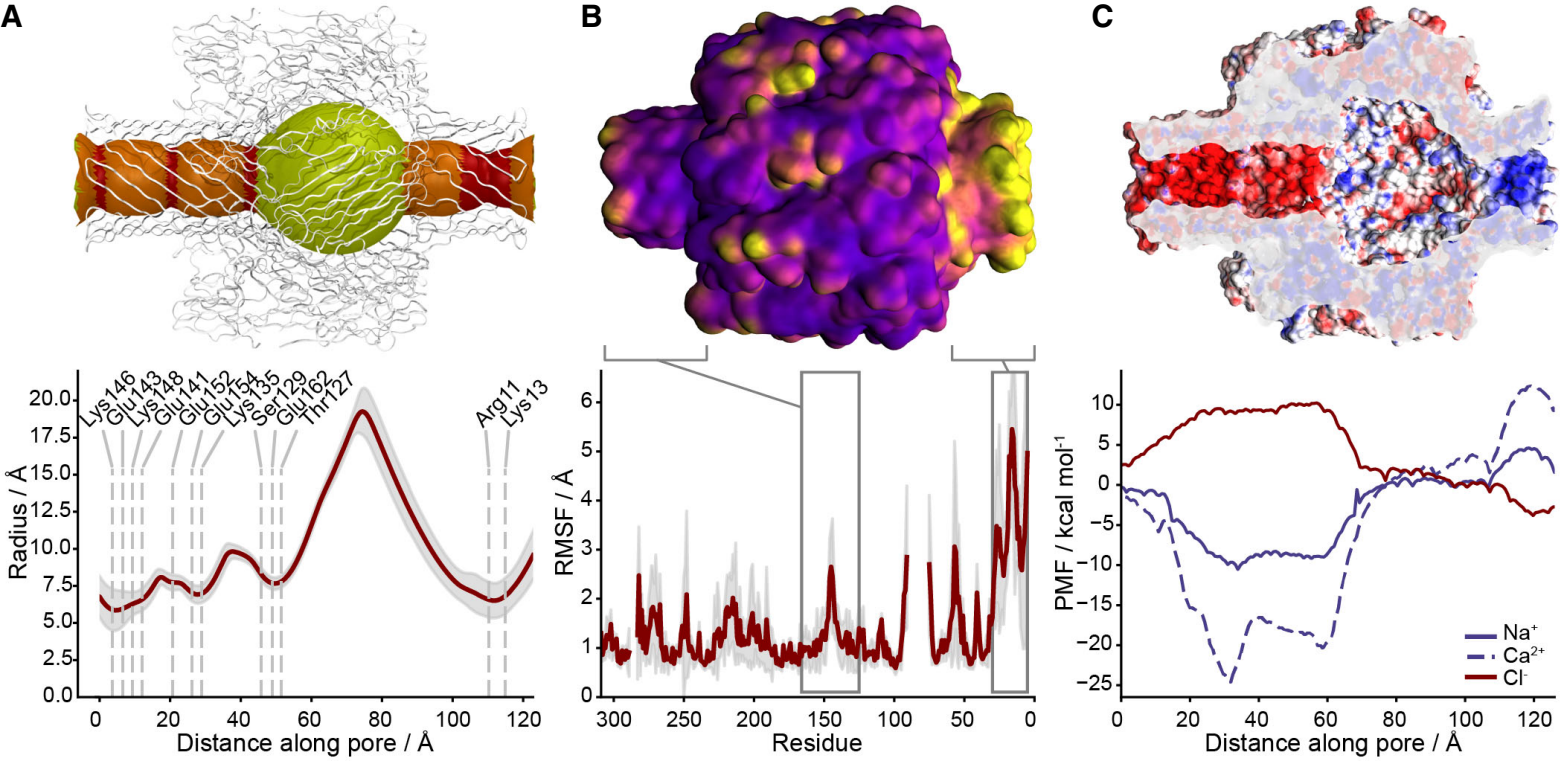
CPB dynamics and NBP measurements Average pore radius in MD simulations. Above, regions with mean radius < 8 Å (constriction points) are shown in red, > 8 Å and < 10 Å in orange, and > 10 Å in yellow. In the graph, mean values are shown in red, and standard deviations in gray. Polar amino acids at constriction points are annotated.
*Clostridium perfringens* β‐toxin (CPB) root mean square fluctuation (RMSF) averaged over the eight CPB chains and three simulation repeats. Above, most mobile regions are shown in yellow, and least mobile in purple. In the graph, mean RMSF values are shown in red, and their standard deviation in gray. The N‐terminal β‐barrel Protrusion (NBP) is the most mobile region and, along with the flexible intracellular mount of transmembrane β‐barrel, is annotated in the graph.Electrostatic properties of the pore internal cavity. Above, negative regions are shown in red and positive ones in blue. In the graph below, potential mean force profiles along the pore axis for Na^+^, Ca^2+^, and Cl^−^ ions are shown. The NBP features a small positive region selective to anions, while the transmembrane β‐barrel is expected to be highly selective to cations.

**Figure EV4:**
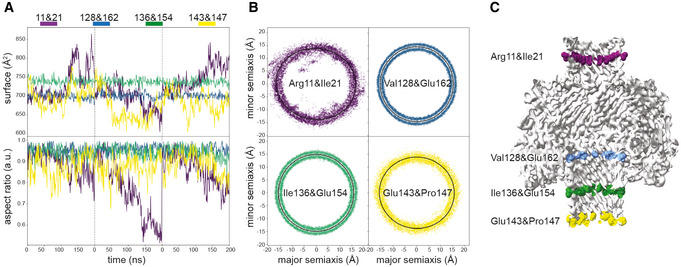
*In silico* flexibility of the CPB channel For each conformation in our *Clostridium perfringens* β‐toxin (CPB) simulation, we extracted the coordinates of Cα at each constriction point and fitted them with an ellipse. The constriction point at the intracellular side of the toxin (residues 143 and 147) is the most dynamic, featuring the largest fluctuations in the fitted ellipse.
Time evolution of constriction point surface area and aspect ratio of three consecutive 200 ns simulations.Position of all extracted Cα coordinates of each constriction point, aligned so that the each fitted ellipse is centered at the origin and oriented so that its major semiaxis is parallel to the x‐axis. Black ellipses fitted to these points represent the average constriction points shape. Only the constriction point at the intracellular side is noticeably elliptical, with Cα atoms featuring larger deviations from the fitted ellipse.Position of the constriction points shown color coded in the cryo‐EM map cross section.

To investigate the mechanical and physical properties of the CPB pore, we carried out atomistic molecular dynamics (MD) simulations of it inserted into a lipid bilayer. The CPB channel features four constriction points in its two β‐barrels (Fig 3A). The narrowest points have a ~ 6 Å mean radius and are located within the NBP at the level of the positively charged Arg11 and Lys13. The channel reaches a maximum radius of about 20 Å within the vestibule of the cap. Quantifying the root mean square fluctuation (RMSF) of the toxin atoms revealed that the most flexible regions are the NBP and the intracellular turns of the transmembrane β‐barrel (Fig 3B). The NBP, while maintaining its overall structure, can oscillate off‐axis, whereas the pore turns can squeeze off a perfectly circular symmetry contributing to reducing the local pore radius (Fig EV4A–C). These observations are consistent with EM data obtained by performing a multibody analysis on the CPB particles and could explain the variability observed previously in the channel conductance (Shatursky *et al*, 2000; Fig EV2F and Movie EV1). Examining charge distribution in the pore lumen revealed a high density of positive charges inside the β‐barrel protrusion due to Arg11 and Lys13 (Fig 3C). Potential mean force (PMF) profiles for Na^+^, Ca^2+^, and Cl^−^ estimated via umbrella sampling indicated that this region should constitute an energy barrier for cations (4.6 kcal/mol for Na^+^). The wide cap vestibule features a balanced amount of positive and negative charges, while the transmembrane β‐barrel is overall negatively charged, especially due to Glu152 and Glu162. These amino acids, associated with constriction points, determine a highly negative PMF for cations (as low as −24.6 kcal/mol for Ca^2+^) and a large 10.2 kcal/mol barrier for Cl^−^ in agreement with previous reports suggesting that the CPB pore is mostly permeable to cations (Shatursky *et al*, 2000; Manich *et al*, 2008).

### Comparison with other hemolysins‐like β‐PFTs

**Figure EV5:**
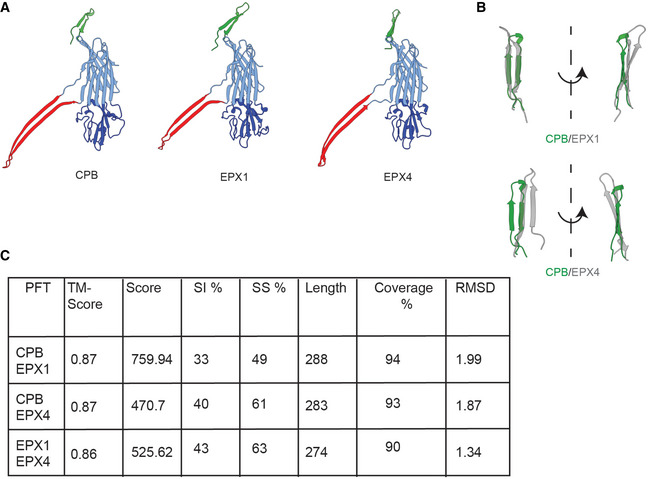
The β‐toxin subfamily of hemolysin‐like β‐pore‐forming toxins (PFTs) Side by side comparison of protomer structures of CPB, EPX1 (PDB: 7T4E), and EPX4 (PDB: 7T4D) color coded as in Fig 1 (NBP—green, cap—light blue, rim—dark blue, and stem—red).A magnified view of the NBP region showing that while CPB and EPX1 share the same NBP fold the EPX4 barrel is reversed.Pairwise structure alignment of the three PFTs shows that they belong to the same subclass of the hemolysin family. The alignments and scores were performed using the RCSB.org analysis using the classic combinatorial extension algorithm (Shindyalov & Bourne, 1998). TMScore denotes the template modeling score (1—perfect match), SI—sequence identity percentage, SS—sequence similarity percentage while the Length denotes the number of residue pairs that are structurally equivalent. CPB, *Clostridium perfringens* β‐toxin; NBP, N‐terminal β‐barrel protrusion; EPX *Enterococcus* pore‐forming toxin.

In this study, we explored the structure of the membrane inserted oligomeric CPB and showed that it belongs to a new subclass of hemolysin‐like β‐PFTs that contain an additional β‐barrel domain in its extracellular side. After our first version of the manuscript was released on a preprint server, eight novel enterococcal hemolysin‐like β‐PFTs were reported (Xiong *et al*, 2022). Interestingly, the structure of two of them was solved and showed that they also adopt an NBP fold atop the cap domain. A structural comparison shows a remarkable structural similarity suggesting that they also belong to the CPB subfamily of hemolysin‐like PFTs (Fig EV5A–C). It is of particular interest that the NBP structure of EPX1 toxin has the same fold as CPB while the NBP of EPX4 toxin is reversed. This supports our mutagenesis results that the NBP β‐barrel structure can be highly flexible. A detailed comparison of the N‐terminal domains with structures of other oligomeric hemolysins is not possible. The N‐terminus of the oligomeric γ‐hemolysin is disordered (Yamashita *et al*, 2011). For NetB, oligomer crystallization was only possible after removing the first 20 amino acids of the protein (Savva *et al*, 2013). For *C. perfringens* δ‐toxin, only the monomer structure is available (Huyet *et al*, 2013)(Savva *et al*, 2013). In δ‐toxin soluble monomer, unlike *S. aureus* Hla, the N‐terminus adopts a β‐hairpin conformation, which extends halfway along the cap β‐sandwich and contacts the pre‐stem (Fig EV3A). Because of the high sequence similarity between CPB and *C. perfringens* δ‐toxin (Appendix Figs S1 and S2), it is reasonable to assume that the N‐terminus of CPB adopts a similar conformation in the water‐soluble monomer. To gain insight into the putative structure of CPB soluble monomer, we made use of recent advances in protein structure prediction (Baek *et al*, 2021; Jumper *et al*, 2021). Both Rosetta and AlphaFold generated predictions similar to the structure of δ‐toxin monomer (Fig EV3C and D) with an N‐terminus folded back and forming a β‐hairpin.

### Putative structural rearrangements of CPB

**Figure 4:**
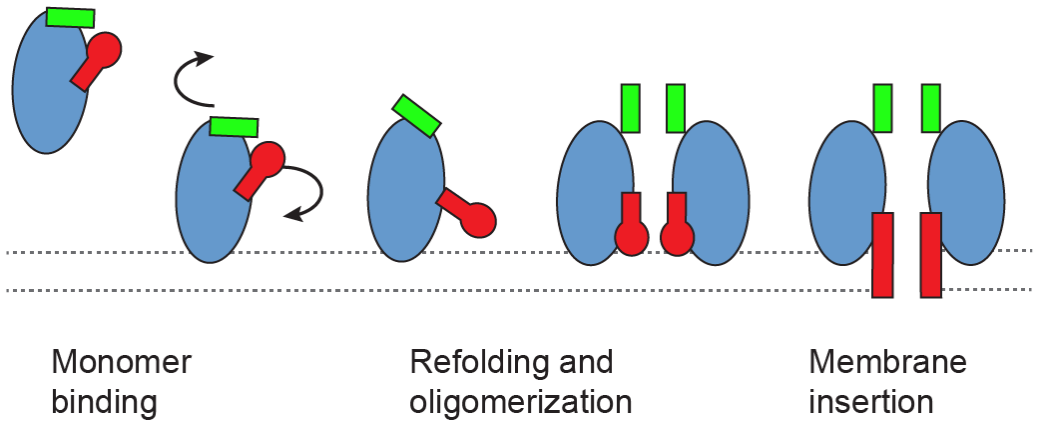
Putative model of CPB mode of action Schematics of the predicted structural changes during *Clostridium perfringens* β‐toxin (CPB) mode of action from the soluble inactive monomer to membrane‐inserted oligomer subunits. Based on high level of sequence conservation with δ‐toxin, we speculated that in soluble CPB the N‐terminal hairpin (green rectangle) is located on top of the cap and rim domains (blue ellipsoid) in contact with the folded pre‐stem domain (red club). We speculate further that the release of the pre‐stem domain and membrane insertion coincides with the reorganization of the N‐terminal hairpin, which through oligomerization and putative pre‐pore formation (Yamashita *et al*, 2014) becomes an N‐terminal β‐barrel.

Comparing the AlphaFold predicted soluble monomer with a CPB protomer suggests putative rearrangements that may occur during oligomerization. In this model, the N‐terminus initially forms an antiparallel β‐sheet folded back against the first β‐strand of the cap domain likely stabilizing the soluble monomer. Following a local concentration increase and oligomerization, we hypothesize that the N‐terminal β‐hairpin swings by 90° and assembles into a β‐barrel while the pre‐stem domain refolds to form the transmembrane β‐barrel (Fig 4 and Movie EV2). The net gain in hydrogen bonds resulting from the barrel formation is similar to the gain due to the N‐terminal latch attachment in Hla. It does not contribute significantly to the stability of the pore, which resists boiling in SDS with or without the NBP (Fig 2A). This contrasts with preliminary results from the enterococcal EPX toxins for which the authors suggest that mutation of the NBP results in decreased stability of the oligomer (Xiong *et al*, 2022). The presence of detergent during the oligomerization of CPB but not EPX could account for this difference. Interestingly, in the predicted δ‐toxin soluble monomer the N‐terminus hairpin covers the subunit contact area at the cap level, which suggests that the CPB N‐terminus may affect folding and oligomerization (Appendix Fig S4A and B). A similar role in regulating oligomerization was also proposed for the N‐terminal domains of the staphylococcal bi‐component leukocidins and Hla (Jayasinghe *et al*, 2006; Yamashita *et al*, 2011). Purification of CPB that lacks the NBP (Δ23CPB) resulted only in very low yields due to aggregation in expressing bacterial cells. It is thus tempting to speculate that the N‐terminal region of CPB plays an additional role in regulating folding of the nascent polypeptide.

In conclusion, the CPB pore has a C8 symmetry unlike the C7 symmetries of the homo‐component members of the hemolysin family and similar to the enterococcal EPX toxins. It is a prototype of a new sub‐group of the hemolysin‐like family of PFTs characterized by a constriction at both ends of the channel forming a bipolar nanopore. The diameter of the two constrictions is smaller than for Hla (Song *et al*, 1996) and similar to the constriction present in the pore formed by MspA, a *M. Smegmatis* porin engineered for nanopore sequencing (Derrington *et al*, 2010). Our PMF profiles indicate that the NBP should constitute an energy barrier for cations while the transmembrane barrel has the opposite effect. Thus, both regions have different selectivity and conduction properties, which together result in the overall selectivity and conduction properties of the whole pore. This asymmetric, bipolar, nature of the CPB channel surface charge is a unique feature that has not been observed in other hemolysin‐like pores. It can be of great interest for nanoengineering applications (Huang *et al*, 2018; Madai *et al*, 2019). The bipolar charge distribution and unique pore architecture of CPB make it an interesting candidate for small molecule sensing, while the double constriction connected by a large cavity could be of interest for selective molecule delivery and transport. In addition, our results suggest that the NBP selectivity can be altered without affecting the pore‐forming ability of the toxin.

Our findings about the CPB pore complex structure provide the basis for further studies on related β‐PFTs. The large number of hemolysin‐like β‐PFTs found in *C. perfringens*, coupled with the known pathogenic role of CPB and the related NetB in necrotic enteritis in different species, suggests that these toxins are important virulence factors for the pathogen. Therefore, they represent valuable targets to develop structure‐based anti‐toxin strategies. The β‐barrel protrusion which defines the new sub‐family of the hemolysins‐like PFTs could also become a novel target for drug design.

## Materials and Methods

### Materials

Chemicals were purchased from Merck (Switzerland) or Sigma‐Aldrich (Switzerland). Detergents were purchased from Anatrace (USA) or Sigma (Switzerland), SMA (SMALP 20010P and SMALP 50005P) from Orbiscope were a kind gift from the SMALP network and Polyscope, and oligonucleotides (Appendix Table S1) were purchased from Microsynth (Switzerland). Pairwise flexible alignment was performed using the FATCAT v2 server (Li *et al*, 2020).

### Molecular cloning

The codon optimized ORF (Genscript) encoding CPB was cloned as N‐terminal His_6_ or N‐terminal GST fusion into pET19‐b (Novagen) using NcoI and BamHI sites. CPB containing a C‐terminal His_6_‐tag was generated using Q5® site‐directed mutagenesis kit (NEB), pET‐19b_His_6__CPB as template and primer pair #1 and elongation time of 4 min to delete the N‐terminal His_6_, followed by a second PCR step using primer pair #2 and elongation time of 4 min to introduce the C‐terminal His_6_. The His_6__CPB_Δ23_ construct was generated using Gibson Assembly^®^ (NEB) and pET‐19b_His_6__CPB was linearized using NcoI and BamHI sites. A PCR product containing His_6_‐CPB_Δ23_ was generated with primer pair #4, Ta and an elongation time of 1 min. CPB chimera constructs were generated using Gibson Assembly^®^ master mix (NEB) and pET‐19b_His_6__CPB was linearized using NcoI and BamHI sites. PCR products containing the codon optimized N‐terminal domains of δ‐toxin (His_6_‐CPB_Δ23_δ‐toxin_(1–24)_), Hla (His_6_‐CPB_Δ23_Hla_(1–20)_) and HlgB (His_6_‐CPB_Δ23_HlgB_(1–19)_) were generated using pET‐19b_His_6__CPB as template and indicated primer pairs, Ta and an elongation time of 1 min (Appendix Fig S1). The resulting sequences were verified by Sanger Sequencing (Microsynth AG) and plasmids were transformed into *E. coli* Dh5α competent cells (NEB) for amplification.

### Recombinant toxin production and purification

The pET‐19b plasmids encoding the CPB constructs were transformed into BL21 (DE3) strain (Sigma) for protein over‐expression. Expression, solubility, and purification trials were done in small volumes of 0.05–0.2 l and upscaled to 3–9 l of LB medium (containing 100 μg/ml ampicillin and 1% glucose) according to the expression levels of the various CPB constructs. Expression cultures were inoculated with 1/50 volume of an overnight preculture grown from multiple colonies. Cultures were incubated at 37°C and shaking at 180 rounds per minute. After growing to an OD_600_ of 0.6–0.8, expression cultures were cooled to 20°C and expression of CPB was induced by addition of 0.5 mM isopropyl‐β‐D‐thiogalactopyranoside (IPTG). After protein expression (8–12 h at 20°C), cells were pelleted 60 min at 3,400 *g* and 4°C and stored at −20°C until protein purification.

Cell pellet was resuspended in lysis buffer (50 mM Tris pH 8, 500 mM NaCl, 10 mM imidazole, 1% TritonX‐100) in the presence of lysozyme (0.2 mg/ml) and protease inhibitors (Sigma) followed by cycle high‐pressure homogenization (LM10 microfluidizer). Lysate was stirred on ice for 30 min in the presence of benzonase (Sigma) followed by two additional cycles of high‐pressure homogenization. After removal of cell debris by centrifugation for 60 min at 50,000 *g*, the supernatant was loaded on 5 ml HiTrap chelating column (GE Healthcare, Germany) running on AKTA prime liquid chromatography system. The column was washed with 50 ml lysis buffer (containing 1% TritonX‐100) and with 50 ml Buffer A (50 mM Tris, pH 8, 150 mM NaCl, 10 mM imidazole, 20–50 mM Na cholate). The protein was eluted with a linear gradient of imidazole (10–500 mM in 50 mM Tris pH 8, 500 mM NaCl, 20 mM Na cholate). Finally, fractions containing the oligomeric protein were dialyzed overnight (20 mM Tris pH 8, 150 mM NaCl, 20 mM Na cholate) and concentrated to 2 mg/ml.

For purification of monomeric CPB, the same *E. coli* strain was used but no detergent was added during the purification steps and a final concentration of 0.4 mg/ml was not exceeded because of protein precipitation due to spontaneous shift to oligomeric form at higher concentrations.

All purification steps were carried out at 4°C. The purity of CPB preparations after each purification step was estimated by denaturing sodium dodecyl sulphate polyacrylamide gel electrophoresis (SDS‐PAGE).

### Preparative size exclusion chromatography

For further analysis by cryo‐EM, preparative size exclusion chromatography (SEC) was applied to separate monomeric CPB and other impurities from oligomers formed in detergent. The pooled and dialyzed protein solution from the metal chelate affinity chromatography was loaded on a 120 ml HiLoad® 16/600 Superdex^®^ 75 pg column (GE Healthcare, Germany). The column was equilibrated in buffer (20 mM Tris, 150 mM NaCl, pH 8) run at a rate of 1 ml/min and eluted in 1 ml fractions. Fractions containing the CPB oligomer were pooled, concentrated, and used for subsequent experiments.

### Sodium dodecyl sulphate polyacrylamide gel electrophoresis

Samples were mixed with 1/5 of their volume of 5× SDS sample buffer and incubated at RT for at least 5 min (or 5 min at 95°C if referred to as boiled) prior to loading on a gel. 10 μl of samples was loaded on 10% or 16% polyacrylamide gels and subjected to electrophoresis at limiting current of 55 mA for 45 min. Gels were stained in a 0.2% Coomassie Brilliant Blue G solution for 20 min or fixed in prefixing solution and stained with the sensitive colloidal staining solution.

### Blue native polyacrylamide gel electrophoresis

The samples (10 μg protein/lane) after a clarifying spin (20,000 *g*, 15 min, 4°C) were mixed with 5× BN sample buffer (2.5% (w/v) Coomassie brilliant blue G‐250, 100 mM Bis‐Tris, 250 mM 6‐aminocaproic acid, 50% Glycerol, pH 7.0) and analyzed by electrophoresis in a blue native gel containing 4–16% gradient of acrylamide (29:1 Acrylamide:Bis) using the SE 600 Vertical electrophoresis system (GE Healthcare Life Sciences). The gels were run at 4°C at constant 200 V for 2 h followed by 6 h at 600 V with max. 20 mA. The mixture NativeMark Unstained Protein Standard (Thermo Fisher) was used to monitor the migration of molecular weight marker proteins. Gels were stained with colloidal Coomassie.

### Negative staining

A 400 mesh carbon‐coated copper grids (CF400‐Cu Electron Microscopy Sciences) were glow discharged using a CCU‐010 sputter/carbon coater (Safematic) with negative polarity (10 mA) for 45 s immediately before usage.A 4 μl of the protein sample was adsorbed to the prepared grids for 45 s. After drying excess liquid with a filter paper (blotting), the samples were washed six times with ddH_2_O and stained immediately by placing the grid on top of a drop of 2% uranyl acetate solution for 1 min. Finally, the stained grid was blotted dry and dried completely under a hood prior analysis. Electron micrographs were recorded at 105′000 x magnification by an Olympus‐SIS Veleta CCD camera using a Tecnai Spirit G2 electron microscope (FEI, USA) operating at an acceleration voltage of 80 kV.

### Sample preparation for cryo‐EM

SMAs are synthetic copolymers composed of styrene (S) and maleic acid anhydride (MA) that function as an alternative to detergents and amphipols (Dorr *et al*, 2016). They can be used to directly purify a membrane protein from their natural lipid environment forming SMA lipid particles (SMALPs) in which the membrane proteins are surrounded by a small disk of lipid bilayer encircled by polymer, similarly to MSP nanodiscs. We tested the SMA (S:MA ratio 2.3:1 and 1.4:1) directly as substitute for detergents Big‐Chaps and sodium cholate, without the addition of lipids. Therefore, SMA at 2.5% (w/v) was incubated with oligomeric CPB at a concentration of around 1.5 mg/ml for 1 h at RT and detergent was removed with Amberlite^®^ XAD^®^‐2 biobeads (Sigma) over night at RT. The formed SMA CPB particles were concentrated to around 4 mg/ml and analyzed by single‐particle cryo‐EM following gel filtration.

### Specimen preparation and data collection

For cryo‐EM, 3 μl of the protein sample at different concentrations was deposited onto a copper grid (quantifoil Cu 200 mesh R2/1, R1.2/1.3) that was glow discharged 10–20″ 10 mA using a Baltzers CTA 010. Vitrification was performed by plunging into liquid ethane in an atmosphere at 4°C and 100% humidity using a Vitrobot Mark IV. Vitrified grids were stored in liquid nitrogen prior to acquisition which was performed on a FEI Tecnai F20 equipped with a Falcon III camera. Images were recorded as stack of frames using FEI EPU automatic data collection with a total dose not exceeding 60 e^−^/Å^2^ and processing was performed in RELION (Zivanov *et al*, 2018; Appendix Fig S5A and B). Acquisition was performed over several days using an in‐house liquid nitrogen filling robot for the side entry holder (Gatan 626) which was set to refill the dewar every 3 h. Post‐acquisition, the recorded movie frames were motion corrected and summed using Motioncor followed by CTF correction using Ctffind (Rohou & Grigorieff, 2015; Zheng *et al*, 2017). A small subset (< 2,000 particles) was selected by hand and an initial 2D classification was used to generate the references used for autopicking 1,874,390 particles. Autopicking was followed by 2D classification of 4× binned particles without image alignment which resulted in an initial subset of 792,433 particles. A new round of 2D classification with image alignment on the initial subset resulted in 382,962 particles which were re‐extracted unbinned. 2D classification of the unbinned data set allowed us to select the best particles suitable for high resolution. The best data set selected contained 260,481 which could be refined to an estimated ~ 4.2 Å resolution as estimated by RELION. Performing the implemented Bayesian polishing step led to an improved resolution of 3.8 Å.

### Model building and refinement

Model building inside the electron density map was based on HlgAB (PDB: 3b07; Yamashita *et al*, 2011) and was performed in Coot and Phenix using real space refine (Table 1; Emsley *et al*, 2010; Liebschner *et al*, 2019). Several models were generated for visualization purposes, the CPB octamer model with outside chains for residues lacking a clear EM density in the map and a complete model where the residues with missing EM density were fitted with the most appropriate rotamer and then inspected in Coot and refined to remove clashes. A third model was generated fitting the missing loops using Phenix implementation for fit loops (Fig EV3B). All images were generated using Chimera, Coot, and PyMol (Pettersen *et al*, 2004; Emsley *et al*, 2010; Schrodinger, 2015).

**Table 1:** Refinement statistics. Cryo‐EM data collection, refinement, and validation statistics.

	#1 CPB
	D_1292117811
	(EMDB‐13876)
	(PDB 7Q9Y)
Data collection and processing	
Magnification	80000
Voltage (kV)	200
Electron exposure (e−/Å^2^)	60
Defocus range (μm)	−1.8 to −3.0
Pixel size (Å)	1.306
Symmetry imposed	C8
Initial particle images (no.)	382962
Final particle images (no.)	260481
Map resolution (Å)	3.8
FSC threshold	0.143
Map resolution range (Å)	3.6–7.5
Refinement	
Initial model used (PDB code)	3B07
Model resolution (Å)	4
FSC threshold	0.143
Model resolution range (Å)	3.8/4.5
Map sharpening *B* factor (Å^2^)	−266
Model composition	
Non‐hydrogen atoms	15840
Protein residues	2232
Ligands	0
*B* factors (Å^2^)	
Protein	266
Ligand	
R.m.s. deviations	
Bond lengths (Å)	0.36 Å
Bond angles (°)	0.54°
Validation	
MolProbity score	4
Clashscore	0
Poor rotamers (%)	0
Ramachandran plot	
Favored (%)	97
Allowed (%)	3
Disallowed (%)	0

### Molecular dynamics

Three independent MD simulation repeats were performed by embedding the atomistic CPB octamer into a POPC bilayer, solvating it with TIP3P water, and charge‐balancing the resulting system with Na^+^ counterions. All simulations were parameterized using the Amber14SB (Maier *et al*, 2015) forcefield with Slipids lipid parameters (Jambeck & Lyubartsev, 2012), and performed with the GROMACS engine (Abraham *et al*, 2015). To equilibrate the systems, the temperature was brought up to 310 K over 1 ns with the V‐rescale themostat (NVT conditions). The pressure was then set to 1 bar over 1 ns (NPT conditions) using the Berendesen barostat. During these first equilibration phases, we promoted the relaxation of the flexible NBP region into a conformation of minimal energy by restraining the distances between atoms expected to form an inter‐chain H‐bond. These restraints were then slowly lifted over 15 ns NPT simulation using the Nose‐Hoover thermostat and Parinnello‐Rahman barostat. Finally, 200 ns free NPT simulations were carried out, and conformations collected every 0.1 ns. During all simulations, electrostatics were calculated with the particle mesh Ewald algorithm, and the LINCS algorithm was used to enable the use of a 2 fs time step. Assessment of internal pore diameter calculations was performed with HOLE2 (Smart *et al*, 1996) on each simulation conformation. The root mean squared fluctuation of each atom was calculated over an aggregation of all independent trajectories, and then cast onto a volume representation to produce the visualization of Fig 3. PMF profiles along the pore z‐axis for Na^+^, Ca^2+^, and Cl^−^ ions were calculated via umbrella sampling. To this end, ions were restrained with a 1,000 kJ nm‐2 harmonic potential along the axis with a 2 Å spacing and simulated at each position for 10 ns. We then reconstructed the one‐dimensional PMF profile along the pore using the weighted histogram analysis method (WHAM). The electrostatics of the internal pore cavity were visualized by casting the continuum electrostatic potential calculated with the adaptive Poisson‐Boltzmann solver (Jurrus *et al*, 2018) onto a simulated volumetric map of CPB. Volumes simulations and image rendering were produced with VMD (Humphrey *et al*, 1996).

### Cytotoxicity assay

The HEK 293FT/CD31‐GFP cells were previously established by lentiviral transduction of HEK 293FT cells with lentiviral plasmid encoding mouse CD31‐GFP (Bruggisser *et al*, 2020) rendering them sensitive for CPB. HEK293FT/CD31‐GFP cell lines were cultured in DMEM medium (Gibco, product 41965‐039) supplemented with 10% fetal calf serum FCS (Gibco), 10 mM Hepes pH 7.2 (Gibco), 4 mM L‐Glutamine (Gibco), in the presence of penicillin–streptomycin (Gibco) and puromycin (1 mg/ml), grown at 37°C in an atmosphere containing 5% CO2.

### Effects of CPB and CPB mutants on cells were measured by using resazurin assay

Cells (2 × 104 cells/ml) grown to confluency in a 96‐well plate were incubated with CPB (1 μg/ml CPB starting concentration, 1:2 dilution steps) for 24 h. Resazurin dye was added to a 0.002% final concentration, incubated for 4 h at 37°C, and fluorescent signal intensity was quantified using the EnSpire Multimode Plate Reader (PerkinElmer) at excitation and emission wavelengths of 540 and 612 nm, respectively.

## Author contributions

**Julia Bruggisser:** Conceptualization; investigation; writing – original draft; writing – review and editing. **Ioan Iacovache:** Conceptualization; investigation; writing – original draft; writing – review and editing. **Samuel C Musson:** Investigation; writing – review and editing. **Matteo T Degiacomi:** Supervision; investigation; writing – review and editing. **Horst Posthaus:** Conceptualization; supervision; project administration; writing – review and editing. **Benoît Zuber:** Conceptualization; supervision; project administration; writing – review and editing.

## Disclosure and competing interests statement

The authors declare that they have no conflict of interest.

## Supporting information




Appendix S1

Expanded View Figures PDF

Movie EV1

Movie EV2

PDF+

## Data Availability

The cryo‐EM data and the associated pdb model from this publication have been deposited to the wwPDB protein data bank (https://www.wwpdb.org/) and assigned the identifier EMD-13876 (http://www.ebi.ac.uk/pdbe/entry/EMD-13876) for the map and PDB ID 7Q9Y for the model (http://www.rcsb.org/pdb/explore/explore.do?structureId=7Q9Y).
