# Supplementary material for: Cryo‐EM structure of the octameric pore of Clostridium perfringens β‐toxin
Source: EMBO Rep. 2022 Oct 10;23(12):e54856. doi: 10.15252/embr.202254856 (PMC9724662; doi:10.15252/embr.202254856)
Supplement: Supplementary file 1 — Appendix S1 [file EMBR-23-e54856-s002.pdf]

Table of Contents:

Appendix Table S1 2
Appendix Figure S1 3
Appendix Figure S2 4
Appendix Figure S3 5
Appendix Figure S4 6
Appendix Figure S5 7

#	Construct	Sequences 5'-3'	T_a [C°]
1	CPB-His_6	**F1:** AACGACATCGGCAAGACC<br>**R1:** CATGGTATATCTCCTTCTTAAAGTTAAAC<br>**F2:** CACCACCACCACTAAGGATCCGGCTGCTAACAA<br>**R2:** GTGGTGGCCGCTACCAATCGCGGTCACTTTGTG	63, 64
2	His_6-$CPB_{\Delta 23}$	**F:** ACTTTAAGAAGGAGATATACATGCACCATCATCACCATCACAACGATAAG<br>CAA<br>ATCATTAGC<br>**R:** TCGGGCTTTGTTAGCAGCCGTTAAATCGCGGTCACTTTG	58
4	His_6-$CPB_{\Delta 23}Hla_{(1-20)}$	**F:** ACTTTAAGAAGGAGATATACATGCACCATCATCACCATCACGCTGATTCA<br>GACATAAATATTAAAACAGGAACGACCGACATCGGTAGCAACACCACCGTGA<br>ACGATAAGCAAATCATTAGC<br>**R:** TCGGGCTTTGTTAGCAGCCGTTAAATCGCGGTCACTTTG	58
5	His_6-$CPB_{\Delta 23}$δ-$toxin_{(1-24)}$	**F:** CTTTAAGAAGGAGATATACATGCACCATCATCACCATCACGGAAGTAATG<br>ATCTAGGGTCAAAATCTGAAATCCGTAAGGAGGAAAACGGTAATGTGACCAT<br>TATCACGCAGAACGATAAGCAAATCATTAGC<br>**R:** CTTTGTTAGCAGCCGTTAAATCGCGGTCACTTTG	58
6	His_6-$CPB_{\Delta 23}HlgB_{(1-19)}$	**F:** CTTTAAGAAGGAGATATACATGCACCATCATCACCATCACGCTGAAGGAA<br>AAATAACACCCGTAAGTGTTAAGAAAGTTGATGACAAGGTGACCCTGAACGA<br>TAAGCAAATCATTAGC<br>**R:** GCTTTGTTAGCAGCCGTTAAATCGCGGTCACTTTG	58

Appendix Table S1

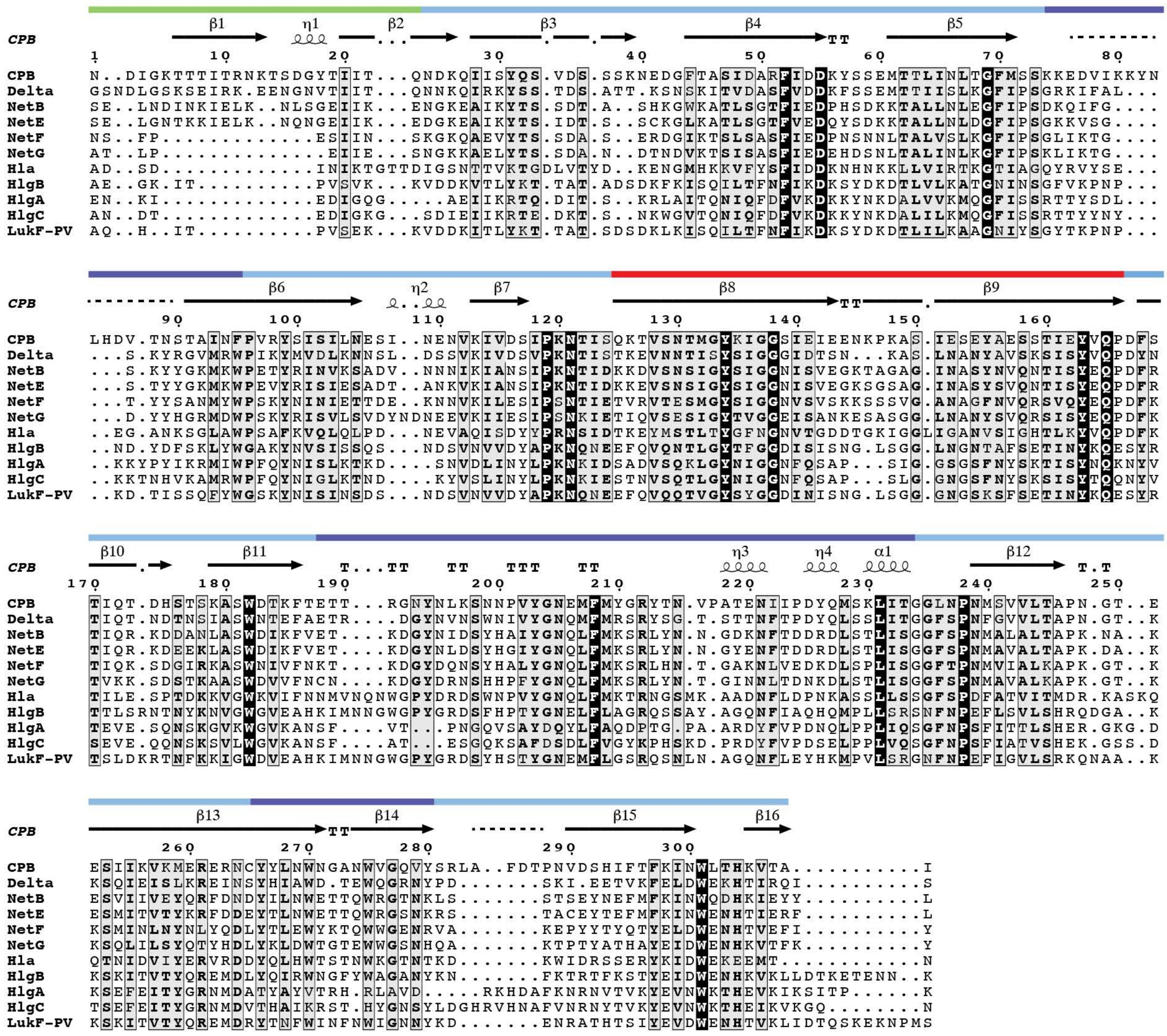

Appendix Figure S1

Sequence Alignment of CPB and related toxins. CPB (Uniprot ID: Q9L403), Delta toxin (Uniprot ID: B8QGZ7), NetB (Uniprot ID: A8ULG6), NetE (Uniprot ID: A0A0D3QGW9), NetF (Uniprot ID: A0A0D3QGV4), NetG (Uniprot ID: A0A0D3QH83), and representative sequences from the S. aureus, including leukocidin S components: HlgA (Uniprot ID: P0A074), HlgC (Uniprot ID: Q07227), F components: LukF (Uniprot ID: Q5FBD2), HlgB (Uniprot ID: P0A077) and hemolysin Hla (Uniprot ID: P09616). Multiple sequence alignments were done using T-coffee and figure has been made using ESPript program. Secondary struture elements (arrows for strands, coils for helices and dotted lines for unstructured) are shown in black at the top for CPB. Different domains are coloured coded as in Figure 1.

	Clostridium						*Enterococcus*		*Staphylococcus*				
									S		F		
	NetB	NetE	NetF	NetG	CPB	Delta	Epx1	Epx4	HlgA	HlgC	LukF	HlgB	Hla
NetB		79	49	51	39	43	33	37	25	22	28	26	29
NetE	79		52	51	41	44	34	39	26	23	31	28	29
NetF	49	52		61	34	40	35	35	25	25	31	26	27
NetG	51	51	61		35	41	38	36	25	23	29	29	30
CPB	39	41	34	35		46	36	41	21	18	27	27	27
Delta	43	44	40	41	46		44	52	26	25	29	29	28
Epx1	33	34	35	38	36	44		43	20	20	27	27	28
Epx4	37	39	35	36	41	52	43		26	23	26	28	27
HlgA	25	26	25	25	21	26	20	26		69	30	31	24
HlgC	22	23	25	23	18	25	20	23	69		30	28	21
LukF	28	31	31	29	27	29	27	26	30	30		72	29
HlgB	26	28	26	29	27	29	27	28	31	28	72		31
Hla	29	29	27	30	27	28	28	27	24	21	29	31	

Appendix Figure S2

Sequence homology between the different hemolysin family members showing CPB highest similarity to the clostridial δ-toxin. Heatmap shows percent identity matrix of protein alignments, colors correspond to the percent identity with high values (red), medium values (white) and low values (blue). The Identity matrix was done using Clustal Omega (Madeira et al., 2019). NetB (Uniprot ID: A8ULG6), NetE (Uniprot ID:A0A0D3QGW9), NetF (Uniprot ID: A0A0D3QGV4), NetG (Uniprot ID: A0A0D3QH83), CPB (Uniprot ID: Q9L403), Delta toxin (Uniprot ID:B8QGZ7), representative sequences from the Entercoccus Epx1 (NCBI Reference Sequence: WP_104660001.1) and Epx4 (NCBI Reference Sequence: WP_053766529.1) and representative sequences from the S. aureus, including leukocidin S components: HlgA (Uniprot ID: P0A074), HlgC (Uniprot ID: Q07227), F components: LukF (Uniprot ID: Q5FBD2), HlgB (Uniprot ID: P0A077) and hemolysin Hla (Uniprot ID: P09616).

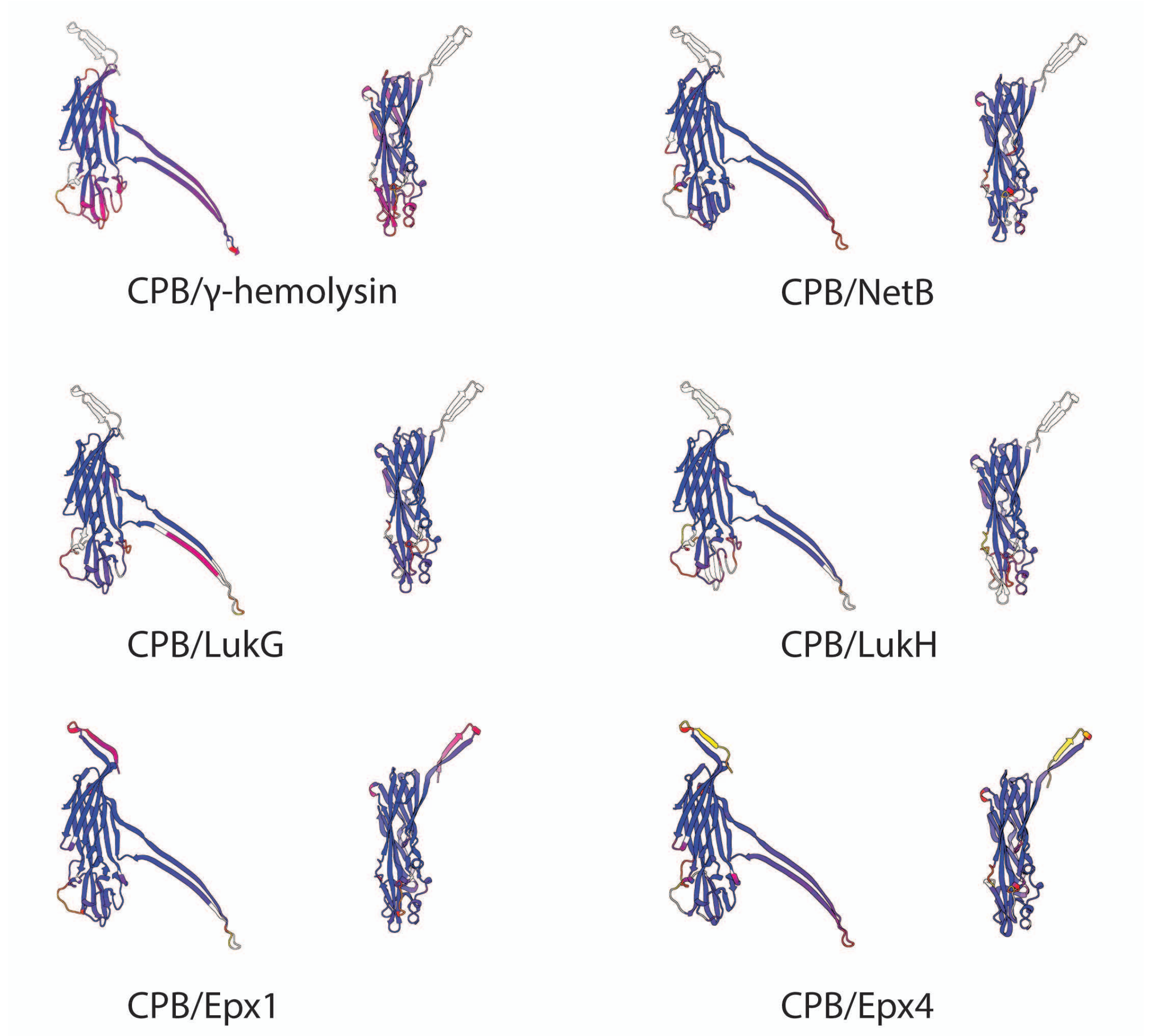

Appendix Figure S3

Pairwise flexible alignment of CPB and different hemolysins represented by a color code on the CPB protomer structure. Blue secondary structure denotes similar protein fold, red and yellow significantly different structures. The NBP together with the rim domain loops show the highest differences between the different proteins of the hemolysin family.

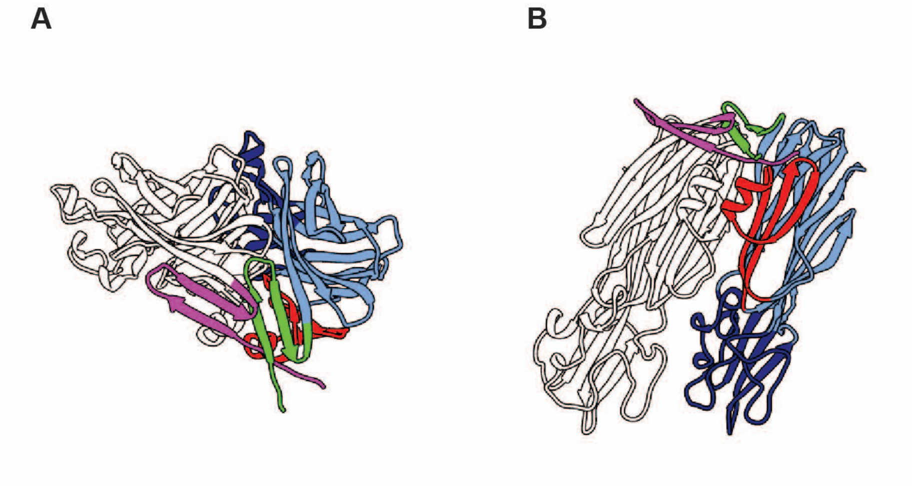

Appendix Figure S4

Top (A) and side (B) view of two AlphaFold predicted soluble CPB monomers positioned as consecutive monomers in the CPB oligomer. One monomer is colored as in Fig. 1 while the second is shown in white with the N-terminus in purple highlighting the clash and overlap at the N-terminal region without extracting the NBP from its position during oligomerization.

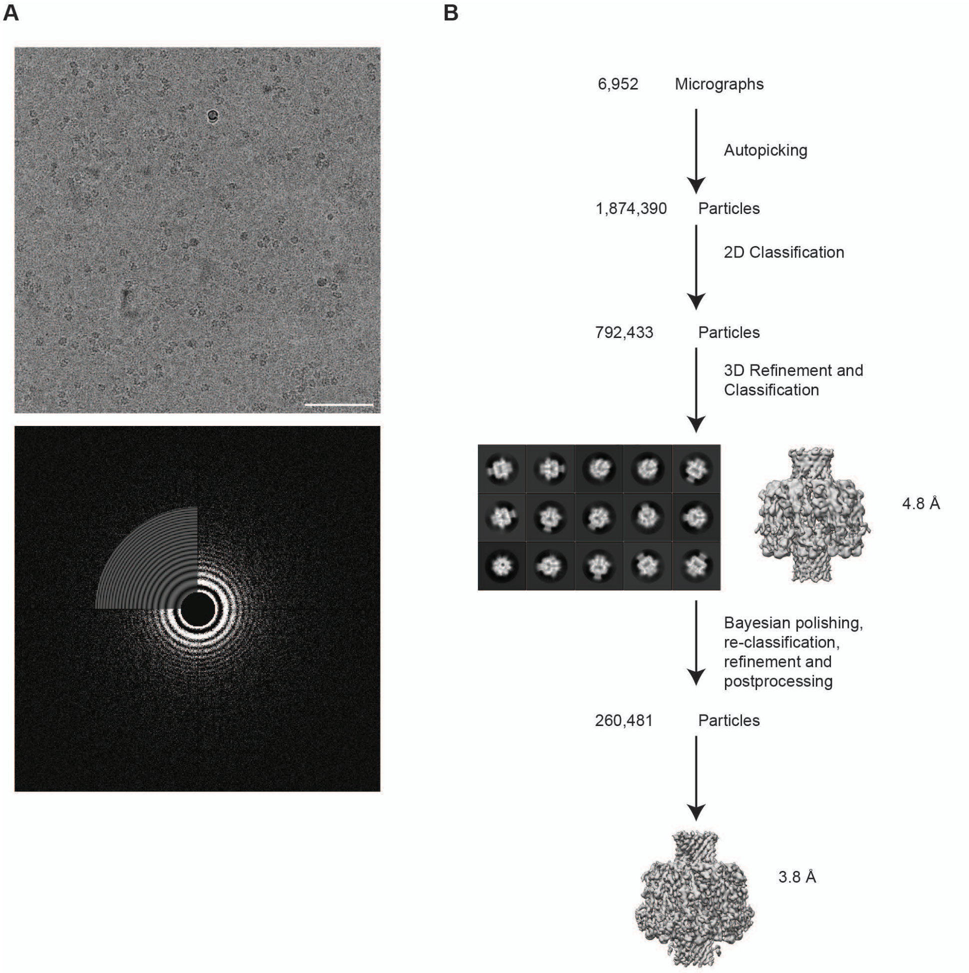

Appendix Figure S5

(a) Example of SMA CPB micrograph and CTF. Scale bar 100nm. (b) Summary of the cryo-EM workflow
