# Supplementary material for: Cryo‐EM structure of the octameric pore of Clostridium perfringens β‐toxin
Source: EMBO Rep. 2022 Oct 10;23(12):e54856. doi: 10.15252/embr.202254856 (PMC9724662; doi:10.15252/embr.202254856)
Supplement: Supplementary file 2 — Expanded View Figures PDF [file EMBR-23-e54856-s005.pdf]

Expanded View Figures

Figure EV1. Oligomerization and solubility of CPB oligomers in different detergents.

A Detergent screen showing cholate and deoxycholate as the best candidates for purification and solubilization of *Clostridium perfringens* β-toxin (CPB) pores. Each detergent was added to the CPB sample for 16 h at 4° and then the sample was centrifuged at 15,000 *g* for 10 min. The pelleted insoluble fraction was recovered in sample buffer and loaded on sodium dodecyl sulphate polyacrylamide gel electrophoresis (SDS-PAGE) together with the soluble fraction and the initial (total) fractions.

B Negative stain of representative CPB oligomers after purification in 30 mM cholate with evenly spread oligomers (top) vs after exchanging cholate with 0.1 mM GDN with aggregates and background particles (bottom).

C, D SDS-PAGE gels of His6-CPB purification in the absence (C) and presence (D) of cholate. 10 µl of supernatant (S), flow through (F), and elution (E) fractions were loaded, and gels stained with Coomassie stain. Most oligomers precipitate in the absence of detergent during the purification, whereas after concentration (C) almost all CPB shifted into the oligomeric state in the presence of cholate.

E Table of the detergents used for the screen, their CMC and their concentration.

F, G Characterization of N- and C-terminal tagged CPB constructs by gel electrophoretic analysis under denaturing (F) and native (G) conditions. SDS-PAGE gel analysis of CPB samples (1 µg per lane) showing monomeric CPB in the absence of detergent and SDS-resistant oligomers in the presence of cholate. CPB samples containing cholate were boiled for 5 min at 95°C (+) or not (−).

© 2022 The Authors

© 2022 The Authors

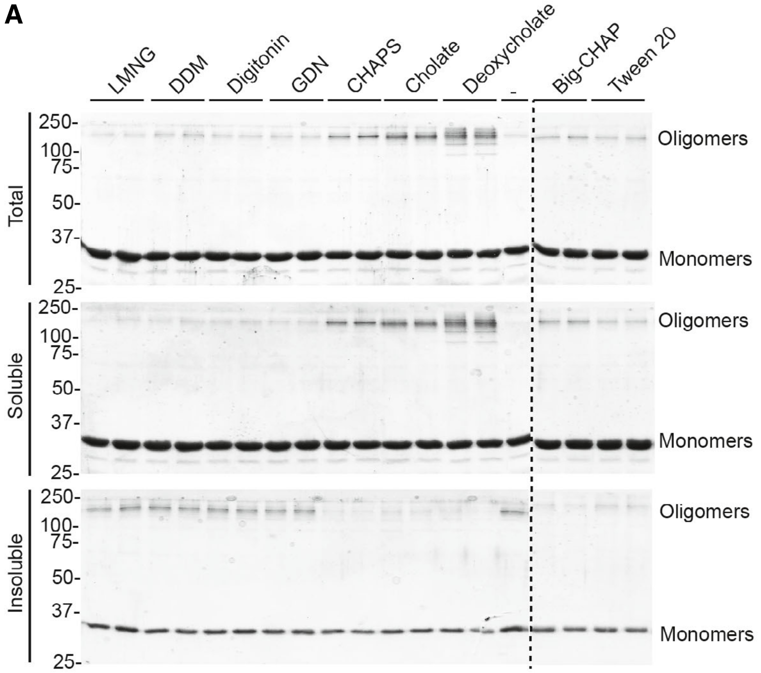

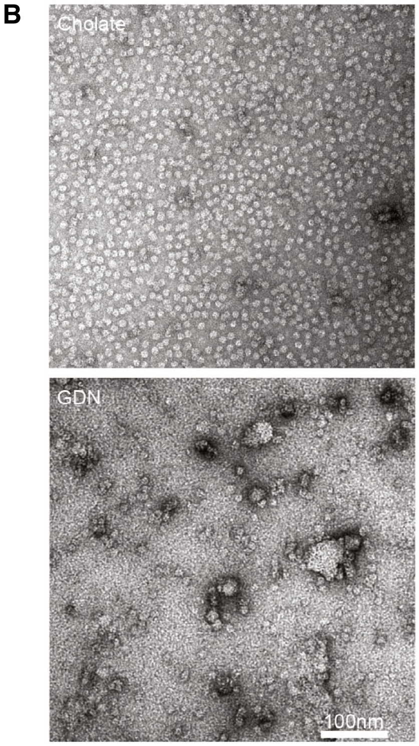

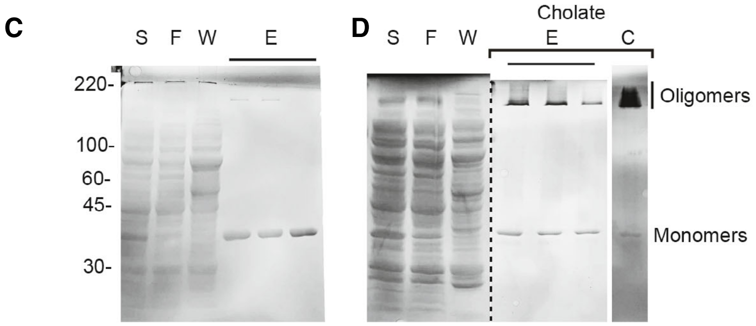

E

Detergent	cmc [mM]	c1 [mM]	c2 [mM]
LMNG	0.001	0.01	0.02
DDM	0.17	1	2
Digitonin	0.4	1	2
GDN	0.018	0.05	0.1
CHAPS	8	15	30
Na Cholate	14	20	40
Na Deoxycholate	5	10	20
Big-CHAP deoxy	1.4	20	6
	0.06	0.6	1.2

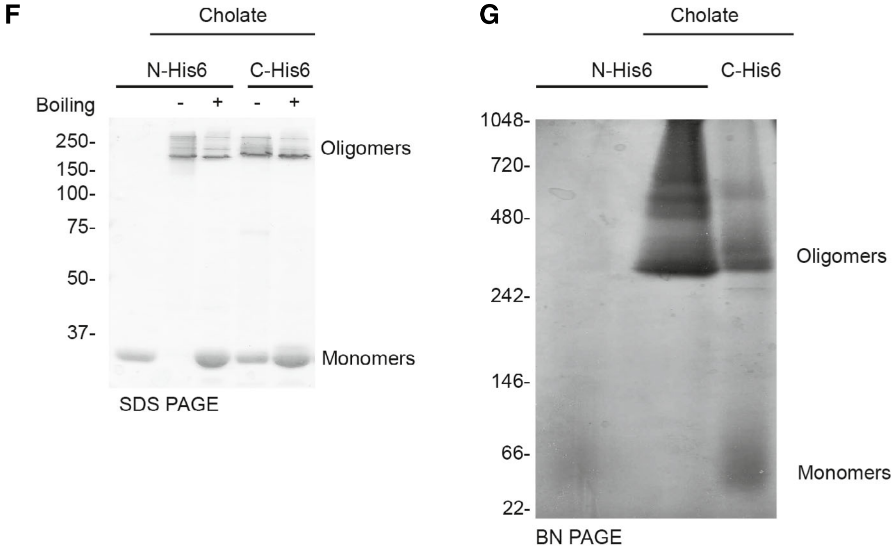

Figure EV1.

© 2022 The Authors

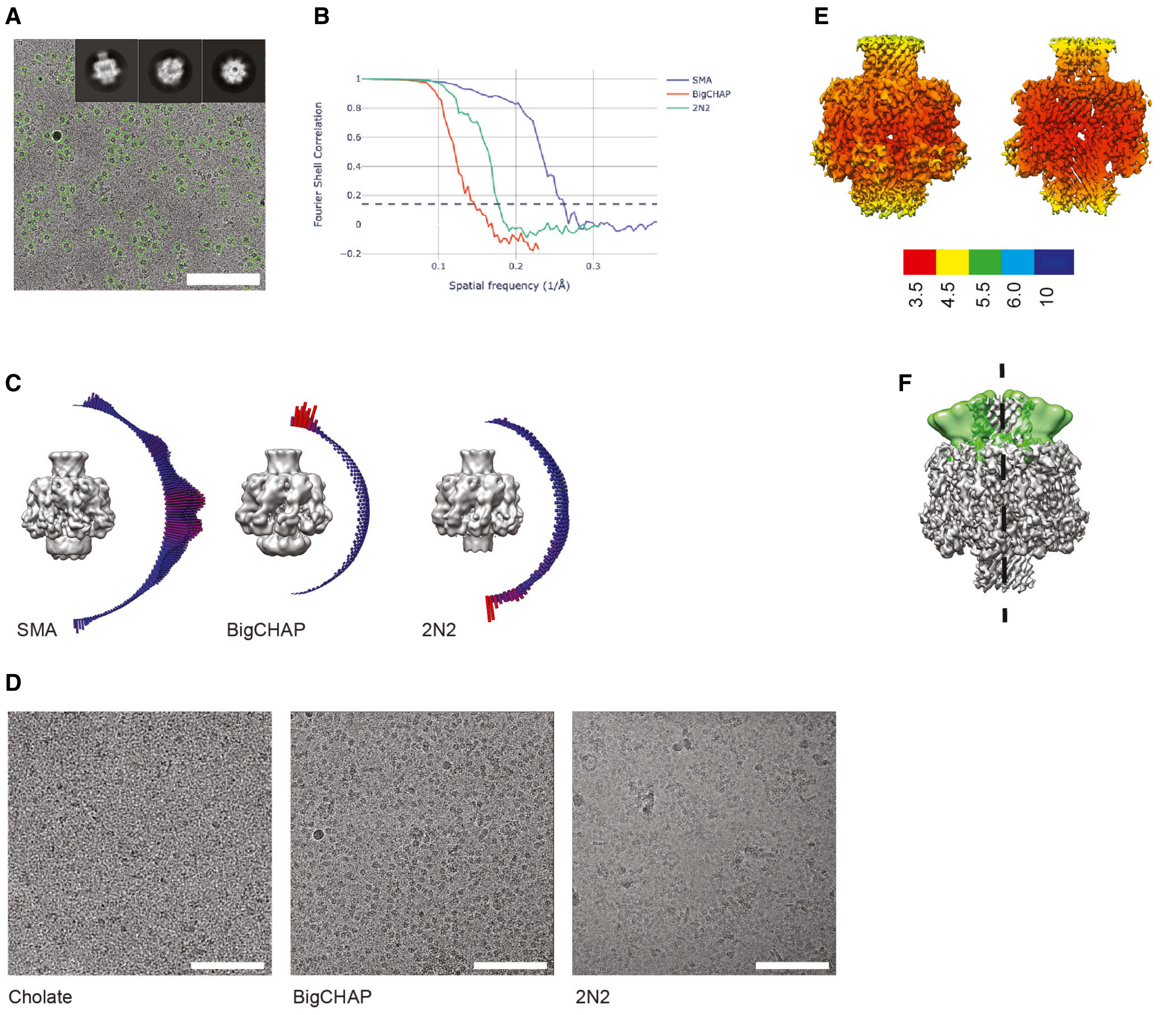

Figure EV2. Cryo-EM of CPB.

A Electron micrograph of a typical field of view of oligomeric *Clostridium perfringens* β-toxin (CPB). Automatically picked particles shown in green. Inset showing characteristic 2D class averages, side, tilted, top. Scale bar is 100 nm.

B FSC showing the resolution of CPB oligomers in different reconstruction conditions with SMA giving the best orientation of the sample on the grid.

C Refined cryo-EM maps and angular distribution of particles of CPB reconstituted in SMA, BigCHAP or 2N2 nanodiscs.

D Representative micrographs of the data in panels B and C.

E Local resolution estimate performed in RELION showing the cap and rim domains at the highest resolution.

F Position of the NBP at the extremes of the multibody analysis performed in RELION (in green) compared to the CPB map (gray).

Figure EV3. Modeling the missing features of the CPB model and structural comparison of CPB and γ-hemolysin.

A Model of *Clostridium perfringens* δ-toxin (2YGT) using the same color code as in Fig 1 showing the position of its N-terminus folded back as an additional strand with the inset showing a comparison between the N-terminus of the δ-toxin (green) and the N-terminus of *Hla* (black—4YHD).

B Model of a protomer of CPB extracted from the oligomer structure color coded as before. The missing loops in the rim domain are modeled and shown in gray. The missing loops are only shown as visual guide for the number of missing amino acids in the model as the map quality in those regions is not good enough for model building.

C, D Prediction of the CPB monomer structure by AlphaFold (C) and RosettaFold (D) color coded as before. The predicted N-terminus folds as the N-terminus of δ-toxin. The missing loops in the rim domains fold similarly to our modeling shown in (B) in the case of alphafold algorithm while RosettaFold modeling extends the longer missing loop into a long β-sheet (D).

E Monomers extracted from the *C. perfringens* β-toxin (CPB) and γ-hemolysin (PDB: 3B07) octamers were aligned (STAMP structural alignment), and the Euclidean distance of their paired Cα atoms measured. In the graph, the black line reports on the distances measured when using our CPB structure. The red line reports on the mean difference when using all structures extracted from our MD simulations for comparison, and the gray shaded region reports on the standard deviation. The protein rendering is colored according to the mean difference of simulated structures.

 © 2022 The Authors

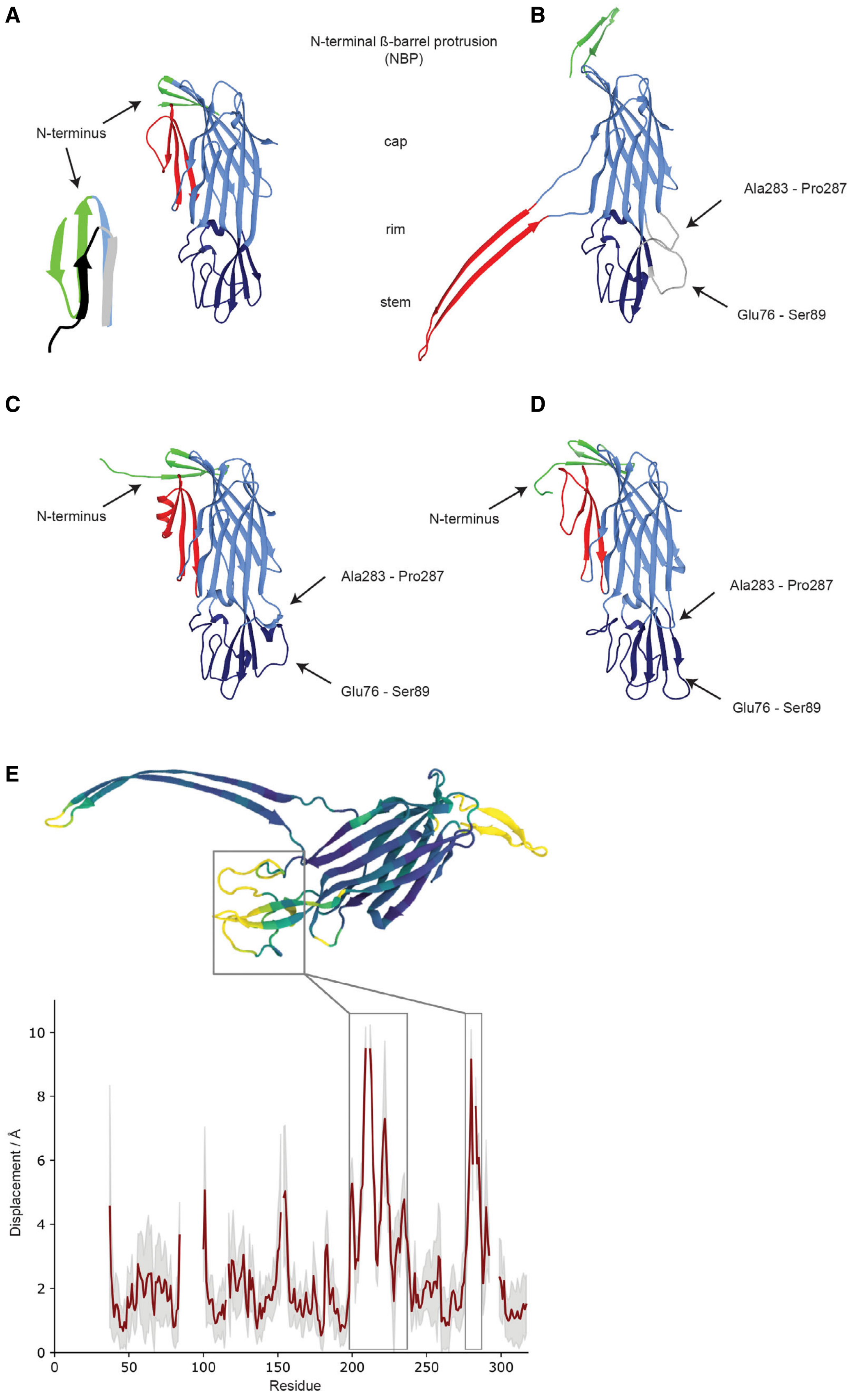
A
B
N-terminal ß-barrel protrusion (NBP)
N-terminus
N-terminus
cap
rim
stem
Ala283 - Pro287
Glu76 - Ser89
C
D
N-terminus
Ala283 - Pro287
Glu76 - Ser89
N-terminus
Ala283 - Pro287
Glu76 - Ser89
E
Displacement / Å
Residue
0
2
4
6
8
10
0
50
100
150
200
250
300

Figure EV3.

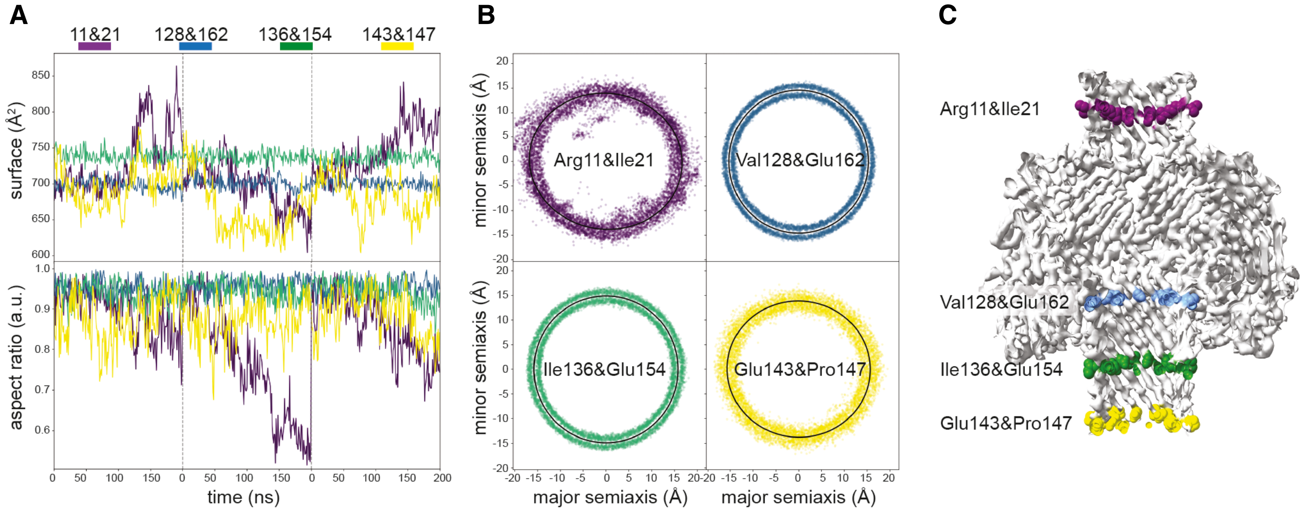

Figure EV4. ***In silico*** **flexibility of the CPB channel.**

For each conformation in our *Clostridium perfringens* β-toxin (CPB) simulation, we extracted the coordinates of Cα at each constriction point and fitted them with an ellipse. The constriction point at the intracellular side of the toxin (residues 143 and 147) is the most dynamic, featuring the largest fluctuations in the fitted ellipse.

A Time evolution of constriction point surface area and aspect ratio of three consecutive 200 ns simulations.

B Position of all extracted Cα coordinates of each constriction point, aligned so that the each fitted ellipse is centered at the origin and oriented so that its major semiaxis is parallel to the x-axis. Black ellipses fitted to these points represent the average constriction points shape. Only the constriction point at the intracellular side is noticeably elliptical, with Cα atoms featuring larger deviations from the fitted ellipse.

C Position of the constriction points shown color coded in the cryo-EM map cross section.

 © 2022 The Authors

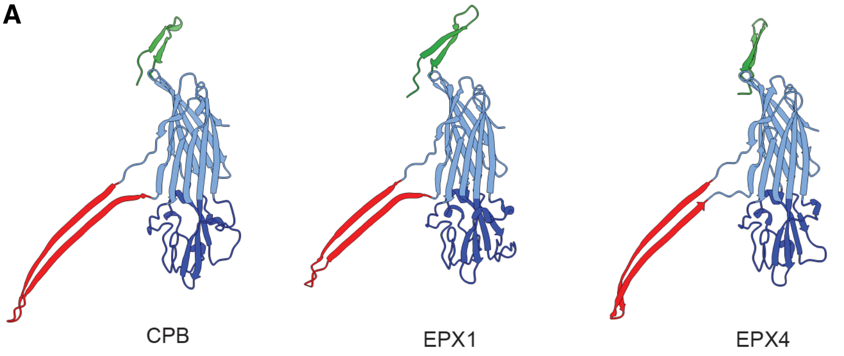

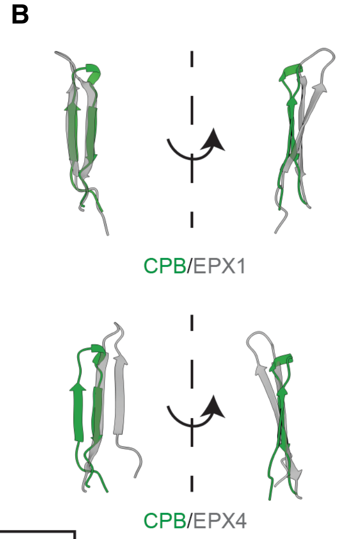

C

PFT	TM-Score	Score	SI %	SS %	Length	Coverage %	RMSD
CPB EPX1	0.87	759.94	33	49	288	94	1.99
CPB EPX4	0.87	470.7	40	61	283	93	1.87
EPX1 EPX4	0.86	525.62	43	63	274	90	1.34

Figure EV5. The β-toxin subfamily of hemolysin-like β-pore-forming toxins (PFTs).

A Side by side comparison of protomer structures of CPB, EPX1 (PDB: 7T4E), and EPX4 (PDB: 7T4D) color coded as in Fig 1 (NBP—green, cap—light blue, rim—dark blue, and stem—red).

B A magnified view of the NBP region showing that while CPB and EPX1 share the same NBP fold the EPX4 barrel is reversed.

C Pairwise structure alignment of the three PFTs shows that they belong to the same subclass of the hemolysin family. The alignments and scores were performed using the RCSB.org analysis using the classic combinatorial extension algorithm (Shindyalov & Bourne, 1998). TMScore denotes the template modeling score (1—perfect match), SI—sequence identity percentage, SS—sequence similarity percentage while the Length denotes the number of residue pairs that are structurally equivalent. CPB, *Clostridium perfringens* β-toxin; NBP, N-terminal β-barrel protrusion; EPX *Enterococcus* pore-forming toxin.

© 2022 The Authors
