# Supplementary material for: Cryo‐EM structure of the octameric pore of Clostridium perfringens β‐toxin
Source: EMBO Rep. 2022 Oct 10;23(12):e54856. doi: 10.15252/embr.202254856 (PMC9724662; doi:10.15252/embr.202254856)
Supplement: Supplementary file 6 [file EMBR-23-e54856-s001.pdf]

EMBO reports

Cryo-EM structure of the octameric pore of *Clostridium perfringens* β-toxin

Julia Bruggisser, Ioan Iacovache, Samuel Musson, Matteo Degiacomi, Horst Posthaus, and Benoît Zuber
DOI: 10.15252/embr.202254856

Corresponding author(s): Benoît Zuber (benoit.zuber@unibe.ch)

Review Timeline:

Submission Date:	14th Feb 22
Editorial Decision:	25th Mar 22
Revision Received:	1st Jul 22
Editorial Decision:	19th Aug 22
Revision Received:	5th Sep 22
Accepted:	14th Sep 22

Editor: Martina Rembold

Transaction Report:

(Note: With the exception of the correction of typographical or spelling errors that could be a source of ambiguity, letters and reports are not edited. Depending on transfer agreements, referee reports obtained elsewhere may or may not be included in this compilation. Referee reports are anonymous unless the Referee chooses to sign their reports.)

1st Editorial Decision 25th Mar 2022

Dear Dr. Zuber

Thank you for the submission of your research manuscript to our journal. We have now received the full set of referee reports that is copied below.

As you will see, the referees acknowledge that the findings are potentially interesting. However, they also point out several technical concerns and have a number of suggestions for how the study should be strengthened, and I think that all of them should be addressed.

Given these constructive comments, we would like to invite you to revise your manuscript with the understanding that the referee concerns (as detailed above and in their reports) must be fully addressed and their suggestions taken on board. Please address all referee concerns in a complete point-by-point response. Acceptance of the manuscript will depend on a positive outcome of a second round of review. It is EMBO reports policy to allow a single round of revision only and acceptance or rejection of the manuscript will therefore depend on the completeness of your responses included in the next, final version of the manuscript.

We realize that it is difficult to revise to a specific deadline. In the interest of protecting the conceptual advance provided by the work, we recommend a revision within 3 months (June 25, 2022). Please discuss the revision progress ahead of this time with the editor if you require more time to complete the revisions.

You can either publish the study as a short report or as a full article. For short reports, the revised manuscript should not exceed 27,000 characters (including spaces but excluding materials & methods and references) and 5 main plus 5 expanded view figures. The results and discussion sections must further be combined, which will help to shorten the manuscript text by eliminating some redundancy that is inevitable when discussing the same experiments twice. For a normal article there are no length limitations, but it should have more than 5 main figures and the results and discussion sections must be separate. In both cases, the entire materials and methods must be included in the main manuscript file.

IMPORTANT NOTE:
We perform an initial quality control of all revised manuscripts before re-review. Your manuscript will FAIL this control and the handling will be DELAYED if the following APPLIES:

1) A data availability section providing access to data deposited in public databases is missing. If you have not deposited any data, please add a sentence to the data availability section that explains that.

2) Your manuscript contains statistics and error bars based on n=2. Please use scatter blots in these cases. No statistics should be calculated if n=2.

When submitting your revised manuscript, please carefully review the instructions that follow below. Failure to include requested items will delay the evaluation of your revision.

When submitting your revised manuscript, we will require:

1) a .docx formatted version of the manuscript text (including legends for main figures, EV figures and tables). Please make sure that the changes are highlighted to be clearly visible.

2) individual production quality figure files as .eps, .tif, .jpg (one file per figure).
Please download our Figure Preparation Guidelines (figure preparation pdf) from our Author Guidelines pages https://www.embopress.org/page/journal/14693178/authorguide for more info on how to prepare your figures.

3) a .docx formatted letter INCLUDING the reviewers' reports and your detailed point-by-point responses to their comments. As part of the EMBO Press transparent editorial process, the point-by-point response is part of the Review Process File (RPF), which will be published alongside your paper.

4) a complete author checklist, which you can download from our author guidelines (). Please insert information in the checklist that is also reflected in the manuscript. The completed author checklist will also be part of the RPF.

5) Please note that all corresponding authors are required to supply an ORCID ID for their name upon submission of a revised manuscript (). Please find instructions on how to link your ORCID ID to your account in our manuscript tracking system in our Author guidelines
()

6) We replaced Supplementary Information with Expanded View (EV) Figures and Tables that are collapsible/expandable online. A maximum of 5 EV Figures can be typeset. EV Figures should be cited as 'Figure EV1, Figure EV2" etc... in the text and their respective legends should be included in the main text after the legends of regular figures.

- For the figures that you do NOT wish to display as Expanded View figures, they should be bundled together with their legends in a single PDF file called *Appendix*, which should start with a short Table of Content. Appendix figures should be referred to in the main text as: "Appendix Figure S1, Appendix Figure S2" etc. See detailed instructions regarding expanded view here:

- Additional Tables/Datasets should be labeled and referred to as Table EV1, Dataset EV1, etc. Legends have to be provided in a separate tab in case of .xls files. Alternatively, the legend can be supplied as a separate text file (README) and zipped together with the Table/Dataset file.

7) Please list the accession numbers and database for the structural data in a formal "Data Availability " section (placed after Materials & Method) that follows the model below (see also < https://www.embopress.org/page/journal/14693178/authorguide#dataavailability>). Please note that the Data Availability Section is restricted to new primary data that are part of this study.

Data availability

The datasets (and computer code) produced in this study are available in the following databases:

- RNA-Seq data: Gene Expression Omnibus GSE46843 (https://www.ncbi.nlm.nih.gov/geo/query/acc.cgi?acc=GSE46843)
- [data type]: [name of the resource] [accession number/identifier/doi] ([URL or identifiers.org/DATABASE:ACCESSION])

Please remember to provide a reviewer password if the datasets are not yet public.

*** Note - All links should resolve to a page where the data can be accessed. ***

8) Figure legends and data quantification:
The following points must be specified in each figure legend:
- the name of the statistical test used to generate error bars and P values,
- the number (n) of independent experiments (please specify technical or biological replicates) underlying each data point,
- the nature of the bars and error bars (s.d., s.e.m.)
- If the data are obtained from n {less than or equal to} 2, use scatter blots showing the individual data points.
Discussion of statistical methodology can be reported in the materials and methods section, but figure legends should contain a basic description of n, P and the test applied.
See also the guidelines for figure legend preparation:
https://www.embopress.org/page/journal/14693178/authorguide#figureformat
- Please also include scale bars in all microscopy images.

9) We would also encourage you to include the source data for figure panels that show essential data. Numerical data should be provided as individual .xls or .csv files (including a tab describing the data). For blots or microscopy, uncropped images should be submitted (using a zip archive if multiple images need to be supplied for one panel). Additional information on source data and instruction on how to label the files are available .

10) Our journal encourages inclusion of *data citations in the reference list* to directly cite datasets that were re-used and obtained from public databases. Data citations in the article text are distinct from normal bibliographical citations and should directly link to the database records from which the data can be accessed. In the main text, data citations are formatted as follows: "Data ref: Smith et al, 2001" or "Data ref: NCBI Sequence Read Archive PRJNA342805, 2017". In the Reference list, data citations must be labeled with "[DATASET]". A data reference must provide the database name, accession number/identifiers and a resolvable link to the landing page from which the data can be accessed at the end of the reference. Further instructions are available at .

11) As part of the EMBO publication's Transparent Editorial Process, EMBO reports publishes online a Review Process File to accompany accepted manuscripts. This File will be published in conjunction with your paper and will include the referee reports, your point-by-point response and all pertinent correspondence relating to the manuscript.

You are able to opt out of this by letting the editorial office know (emboreports@embo.org). If you do opt out, the Review Process File link will point to the following statement: "No Review Process File is available with this article, as the authors have chosen not to make the review process public in this case."

We would also welcome the submission of cover suggestions, or motifs to be used by our Graphics Illustrator in designing a cover.

I look forward to seeing a revised form of your manuscript when it is ready and please let me know if you have questions or comments regarding the revision.

Yours sincerely,

Martina Rembold, PhD
Senior Editor
EMBO reports

****

Referee #1:

This manuscript reports the long awaited PFT structure of C. perfringens β-toxin. The cryo EM 3.8 Å resolution structure of the toxin in its pore form is beautiful to see although it is largely similar to structures previously observed in members of the hemolysin-like family of β-PFTs. The major point of difference is an N-terminal extension that forms a 16 stranded β-barrel called the N-terminal β-barrel protrusion or NBP for short. The structural analysis suggests the NBP could be functionally important. Firstly, sequence alignments suggest some other family members may adopt similar NBPs in their pore state leading to the suggestion of a new sub-group within the wider hemolysin family. Secondly, the NBP forms the narrowest restriction of the entire channel with the diameter as its narrowest point of ~6 Å. Furthermore, the channel is lined with positively charged residues suggesting the toxin pore would be selective for anions. Surprisingly, the transmembrane pore has a high number of negative charges, which is consistent with published data suggesting the toxin pore is cation selective. The charge distribution along the length of the channel is a puzzle and suggests the NBP does not play a role in channel selectivity? The statement in the Discussion that anion binding in the NBP channel could increase flux of cations needs further clarification (Page 6, line 4ff). Thirdly, the formation of the NBP β-barrel might be expected to stabilize the oligomer. However, deletion mutagenesis studies described in the manuscript indicate the NBP does not contribute to pore formation or to stability as measured by boiling in SDS which is not affected by the presence of the NBP. The authors speculate that a role of the NBP is in regulating oligomerization but not all family members are predicted to form the β-barrel N-terminal extension. Surprisingly, the authors find the presence of an NBP does influence cytotoxic activity. Is the receptor binding site location known for the toxin? Is it possible that the NBP plays a role in recognition of the receptor?

I have a few suggestions to improve the appeal of the work. There is quite a bit of space devoted to the toxin being a potential therapeutic target in the Introduction but no comments in the Discussion how the structure might contribute to therapeutic development. Is the toxin a validated drug target? There is an opportunity to briefly highlight PFTs found in other organisms, particularly humans (MACPFs, complement proteins, gasdermins, Bcl family) on page 2 (line 6: "beyond bacterial infections"). On the same page a comment could be made about a number of papers on nanopore applications of the aerolysin family (line 9).

Overall, this work will be of great interest to the PFT field and provides new mysteries to be solved in the future, particularly the role of NBP and whether the NBP can be modified or replaced to affect activity for nanotech applications.

Minor issues:
(i) Page 1, lines 36: most, but not all, PFTs are secreted as monomers. The aerolysin family are secreted as dimers.
(ii) Page 3, line 28: should use the English spelling of "tryptophan".
(iii) Page 4, line 3: "72.9 nm3". I assume this is a volume measurement so the "3" should be superscripted. Given the previous sentences discuss distances in Å units it should be made clearly stated this is a volume and better to use the same units as the distances (ie. Å).
(iv) Page 6, line 4: should read "result" (ie. singular)
(v) Please add the database identifiers in Table 1.

Referee #2:

Summary:
The manuscript titled Cryo-EM structure of the octameric pore of Clostridium perfringens β-toxin by Bruggisser J. et al., reports the first structure on the CPB toxin oligomer from the human pathogen C. perfingens. In this study, the authors screened multiple detergents to find conditions for cryoEM structural determination of the CPB oligomer. They describe an octameric beta barrel pore structure of the hemolysin-like family of beta pore forming toxins, except with an additional N-terminal beta barrel on top, extending the central channel of the pore. The authors investigated the function of the novel N-terminal domain of CPB toxin, demonstrating that it is dispensable for oligomeric assembly, yet critical for toxicity on HEK cells. The authors also investigated

the channel lumen describing its opposing charge distribution suggesting the pore is permeable to cations. Finally the authors use molecular dynamic simulations to show that the upper beta barrel and the mouth of the transmembrane beta barrel have the most flexibility in the oligomer.
The CPB oligomeric structure is novel and interesting, however, some novelty is lost, concerning the N-terminal beta barrel region, due to the very recent publication of Enterococcus pore-forming (Epx) toxins, which contain a similar Beta barrel structure in Xiong et al., Cell 185:1-15. 2022. The authors should rewrite their findings in the context of these recent structures. Furthermore the authors make claims which could be further supported with experimental evidence in the manuscript. The authors should add more information on the cryoEM methods to improve clarity and condense figures to present new data in the results section. Overall, the structure reported in this manuscript is of importance to researchers in the field of toxin biochemistry, bacterial pathogenesis, and microbiology. With some modifications and additions, this manuscript should be considered for publication in EMBO reports.

Major Points
#1 The authors describe flexible loops Glu76-Ser89, Ala283-Pro287 that may be a binding surface for a protein receptor as reported in their previous paper Brussiger et al., 2020 Cell Host Microbe. If the authors wish to include this important point, they should provide experimental evidence (ex. mutations to these residues, cell toxicity assays) supporting the hypothesis that this region of CPB is responsible for receptor binding.
#2 Overall, the cryoEM workflow on multiple samples is not clear to the reader. Please add a supplemental figure showing the cryoEM workflow including samples, processing steps, and particle numbers. Please provide brief details in the method section on which and how the different datasets were collected. In Supp. Fig. 3. please add example micrographs of each dataset/sample condition (SMA, cholate etc.) collected with representative class averages.
#3 Please rewrite the beginning discussion section to compare CPB with recent structures of Epx toxins in Xiong et al., Cell 185:1-15. 2022.
#4 The authors claim the N-terminal beta barrel fold is responsible for efficient oligomerization of the CPB pore. Their NBP deletion data suggest it has a limited role in oligomerization. Please address this, for example, quantifying the ratio of monomer/oligomer in the N-terminal deletion mutant versus WT in presence or absence of detergent.
#5 Intriguingly the authors report that deletion of NBP abolishes cell toxicity. Please elaborate more in the discussion about potential mechanisms for this observation in light of charge modification in the N-termini in Epx pores in Xiong et al., Cell 185:1-15. 2022.
#6 The authors claim that the CPB oligomer represents the membrane-inserted pore form of the toxin. Could you please discuss previously-characterized pre-pore intermediate states of homolo-gous toxins (ex. Yamashita, D., et al., Nat Commun 5, 4897,2014) to support this statement.
#7 The figures could be reworked and combined to improve flow and clarity. The gels in Supp. Fig. 6 could be combined with Figure 2. New data (i.e. Alphafold structures of monomeric CPB) should be presented earlier, in the results section. Figure 4 should be reworked to better illustrate the proposed model for CPB pore formation. It is confusing because monomers already appear to be dimerizing and prepore to membrane pore insertion is not addressed in the text or figure legend.

Minor Points
Page 1, Line 27: "PFTs s" - extra s
Page 2, Line 4: "mechanisms used PFTs" - missing by
Page 3, Line 10: "this allowed to" - missing noun
Page 3 Line 15: "the members of the hemolysin, the CPB..." - missing -like family
Page 3 Line 20: "beta barrel protrusion" - rename to "top" or mention top from Xiong et al., 2022 Cell paper.
Page 3, Line 26: "position 210" - Please label the residue in figure.
Page 3, Line 28: "tryptophane"- extra e
Page 4, Line 25: "intracellular mouth" - intracellular is confusing, can a better name be used?
Page 4, Line 30: "Supplementary Fig. 3D" - change to 3E
Table 1: missing refinement statistics in table
Supplementary Table 1: "Sequence alignment Sequence alignment" - repeated twice. Citation for-matting in legend is not consistent (DOI). Please overlay CPB secondary structure on top of align-ment in the figure. Please add channel naming (rim, cap etc.)
Supplementary Figure 2: missing celsius. formatting for x g (check all instances throughout manu-script).
Supplementary Figure 2: Please provide a micrograph of negatived-stained SMA oligomers.
Supplementary Figure 3: "putative rearrangements required...." - Missing sub panel F in figure?
Supplementary Figure 3d: Please show a cross section of the resolution showing interior channel.
Supplementary Figure 4: Please report the color code in the legend. Black line in the graph is un-clear or missing.
Supplementary Figure 4: "Nterminus" - Fix with N-terminus.
Supplementary Figure 7: Please add in legend or on graph, which the residues correspond to which constriction point in the channel.

Dear Dr Rembold

Please find below our point-by-point answer to the referees. The time that we needed to prepare our revised manuscript was due to a large number of attempts to produce NBP-less mutant toxins in large quantity in order to address one referee #2 request #4. In spite of our efforts, we could not produce it in sufficient amount. This very fact actually hints that the NBP is necessary for proper folding and/or stability of the toxin monomer and thereby answers the referee question. We have mentioned this in the manuscript.

Thank you for your consideration.
Kind regards
Benoît Zuber

Referee #1:

This manuscript reports the long awaited PFT structure of C. perfringens β-toxin. The cryo EM 3.8 Å resolution structure of the toxin in its pore form is beautiful to see although it is largely similar to structures previously observed in members of the hemolysin-like family of β-PFTs. The major point of difference is an N-terminal extension that forms a 16 stranded β-barrel called the N-terminal β-barrel protrusion or NBP for short. The structural analysis suggests the NBP could be functionally important. Firstly, sequence alignments suggest some other family members may adopt similar NBPs in their pore state leading to the suggestion of a new sub-group within the wider hemolysin family. Secondly, the NBP forms the narrowest restriction of the entire channel with the diameter as its narrowest point of ~6 Å. Furthermore, the channel is lined with positively charged residues suggesting the toxin pore would be selective for anions. Surprisingly, the transmembrane pore has a high number of negative charges, which is consistent with published data suggesting the toxin pore is cation selective. The charge distribution along the length of the channel is a puzzle and suggests the NBP does not play a role in channel selectivity? The statement in the Discussion that anion binding in the NBP channel could increase flux of cations needs further clarification (Page 6, line 4ff). Thirdly, the formation of the NBP β-barrel might be expected to stabilize the oligomer. However, deletion mutagenesis studies described in the manuscript indicate the NBP does not contribute to pore formation or to stability as measured by boiling in SDS which is not affected by the presence of the NBP. The authors speculate that a role of the NBP is in regulating oligomerization but not all family members are predicted to form the β-barrel N-terminal extension. Surprisingly, the authors find the presence of an NBP does influence cytotoxic activity. Is the receptor binding site location known for the toxin? Is it possible that the NBP plays a role in recognition of the receptor?

We thank the referee for their appreciation of our work and their constructive comments. We have re-written the discussion about the channel and the NBP. In particular, we have removed the statement that anion binding in the NBP could increase flux of cation since it was confusing. In addition we have added comparisons with the newly characterized enterococcal toxins that belong to the same sub-family of hemolysins. The loss of cytotoxic CPB activity in the absence of the NBP is likely in part due to lower solubility of the

monomeric toxin. Whether the NBP plays a role in receptor recognition or not is out of the scope of this manuscript. However, we aim to study the receptor binding of CPB and identify the exact loops and residues involved in binding as part of a follow-up study.

I have a few suggestions to improve the appeal of the work. There is quite a bit of space devoted to the toxin being a potential therapeutic target in the Introduction but no comments in the Discussion how the structure might contribute to therapeutic development. Is the toxin a validated drug target? There is an opportunity to briefly highlight PFTs found in other organisms, particularly humans (MACPFs, complement proteins, gasdermins, Bcl family) on page 2 (line 6: "beyond bacterial infections"). On the same page a comment could be made about a number of papers on nanopore applications of the aerolysin family (line 9).

Yes, CPB toxin is a validated drug target as clearly shown in Uzal et al 2014 (cited in the manuscript). We have rearranged and added comments to the discussion section to improve the appeal of the work as kindly suggested by the referee.

Overall, this work will be of great interest to the PFT field and provides new mysteries to be solved in the future, particularly the role of NBP and whether the NBP can be modified or replaced to affect activity for nanotech applications.

Minor issues:
(i) Page 1, lines 36: most, but not all, PFTs are secreted as monomers. The aerolysin family are secreted as dimers.
Corrected as suggested
(ii) Page 3, line 28: should use the English spelling of "tryptophan".
Corrected
(iii) Page 4, line 3: "72.9 nm3". I assume this is a volume measurement so the "3" should be superscripted. Given the previous sentences discuss distances in Å units it should be made clearly stated this is a volume and better to use the same units as the distances (ie. Å).
Corrected
(iv) Page 6, line 4: should read "result" (ie. singular)
Corrected
(v) Please add the database identifiers in Table 1.
Added as suggested

Referee #2:

Summary:
The manuscript titled Cryo-EM structure of the octameric pore of Clostridium perfringens β-toxin by Bruggisser J. et al., reports the first structure on the CPB toxin oligomer from the human pathogen C. perfingens. In this study, the authors screened multiple detergents to find conditions for cryoEM structural determination of the CPB oligomer. They describe an octameric beta barrel pore structure of the hemolysin-like family of beta pore forming toxins, except with an additional N-terminal beta barrel on top, extending the central

channel of the pore. The authors investigated the function of the novel N-terminal domain of CPB toxin, demonstrating that it is dispensable for oligomeric assembly, yet critical for toxicity on HEK cells. The authors also investigated the channel lumen describing its opposing charge distribution suggesting the pore is permeable to cations. Finally the authors use molecular dynamic simulations to show that the upper beta barrel and the mouth of the transmembrane beta barrel have the most flexibility in the oligomer.
The CPB oligomeric structure is novel and interesting, however, some novelty is lost, concerning the N-terminal beta barrel region, due to the very recent publication of Enterococcus pore-forming (Epx) toxins, which contain a similar Beta barrel structure in Xiong et al., Cell 185:1-15. 2022. The authors should rewrite their findings in the context of these recent structures. Furthermore the authors make claims which could be further supported with experimental evidence in the manuscript. The authors should add more information on the cryoEM methods to improve clarity and condense figures to present new data in the results section. Overall, the structure reported in this manuscript is of importance to researchers in the field of toxin biochemistry, bacterial pathogenesis, and microbiology. With some modifications and additions, this manuscript should be considered for publication in EMBO reports.

We thank the referee for their critical reading and suggestions. We have addressed all the suggestions presented in the new version of the manuscript. The referee implies that novelty has been lost because of the recent paper by Xiong et al. We would like to argue that the first version of our manuscript was posted as a preprint on biorxiv on 23 Nov 2021 (https://doi.org/10.1101/2021.11.23.469684). This precedes the publication of Xiong et al. paper, for which no preprint was released. We have of course referenced extensively the new structures presented by Xiong et al.

Major Points

#1 The authors describe flexible loops Glu76-Ser89, Ala283-Pro287 that may be a binding surface for a protein receptor as reported in their previous paper Brussiger et al., 2020 Cell Host Microbe. If the authors wish to include this important point, they should provide experimental evidence (ex. mutations to these residues, cell toxicity assays) supporting the hypothesis that this region of CPB is responsible for receptor binding.

We do not claim that the loops are definitely the binding site for the receptor but only indicate that it may possibly be it. In order to support the hypothesis, we have added a sequence-structure comparison with known α-hemolysin family members in Appendix Figure S4 showing that the structure of the loops is one of the most diverging parts of the protein hemolysin fold. Some of the homologous toxins have their receptor binding site in this region. In addition, we have moved this part in the discussion and and tuned it down. We now only say that the observations raise the question of whether the loops are in binding to the receptor. Experimental mapping of the receptor binding site is out of the scope of the present manuscript and will be the topic of a follow-up study.

#2 Overall, the cryoEM workflow on multiple samples is not clear to the reader. Please add a supplemental figure showing the cryoEM workflow including samples, processing steps, and particle numbers. Please provide brief details in the method section on which and how the

different datasets were collected. In Supp. Fig. 3. please add example micrographs of each dataset/sample condition (SMA, cholate etc.) collected with representative class averages.

The cryo-EM workflow has been added in the Appendix Figure S3 along with micrographs and classes. In addition micrographs of each dataset are now shown in Figure EV2.

#3 Please rewrite the beginning discussion section to compare CPB with recent structures of Epx toxins in Xiong et al., Cell 185:1-15. 2022.

We have added extensive comparisons to the recent Epx toxins in the manuscript as well as the Appendix and Extended View figures.

#4 The authors claim the N-terminal beta barrel fold is responsible for efficient oligomerization of the CPB pore. Their NBP deletion data suggest it has a limited role in oligomerization. Please address this, for example, quantifying the ratio of monomer/oligomer in the N-terminal deletion mutant versus WT in presence or absence of detergent.

Unfortunately, unlike wild-type CBP, the NBP mutants are very difficult to purify in large quantities in absence of detergent. This indicates that NBP is needed to stabilize the monomeric protein, rather than to promote oligomerization. We have now highlighted this in the text. While the amounts are barely sufficient for cellular studies, they are not enough for a oligomerization study. We do not know why we are able to purify the protein easier in detergent (though in much reduced quantity compared to the WT).

#5 Intriguingly the authors report that deletion of NBP abolishes cell toxicity. Please elaborate more in the discussion about potential mechanisms for this observation in light of charge modification in the N-termini in Epx pores in Xiong et al., Cell 185:1-15. 2022.

We have now addressed this in the text. The data presented in Xiong et al. for the NBP mutants is rather preliminary. The author claim that the Epx NBP mutant toxins form less SDS-resistant oligomers based on gels in their figure S5E but the gels they show do not support this claim. Based on the quite high level of impurity present in the lanes of the NBP mutants, we could speculate that Xiong et al. faced protein solubility issues not unlike our issues when purifying in the absence of detergent. The origin of the decreased toxicity in the Epx mutants is therefore unclear at this stage. It will be interesting to address this in a future study.

#6 The authors claim that the CPB oligomer represents the membrane-inserted pore form of the toxin. Could you please discuss previously-characterized pre-pore intermediate states of homolo-gous toxins (ex. Yamashita, D., et al., Nat Commun 5, 4897,2014) to support this statement.

We have referenced the putative prepore structure in the Figure 4 where the model is presented. We thank the reviewer for pointing out this omission.

#7 The figures could be reworked and combined to improve flow and clarity. The gels in

Supp. Fig. 6 could be combined with Figure 2. New data (i.e. Alphafold structures of monomeric CPB) should be presented earlier, in the results section. Figure 4 should be reworked to better illustrate the proposed model for CPB pore formation. It is confusing because monomers already appear to be dimerizing and prepore to membrane pore insertion is not addressed in the text or figure legend.

Figures and Supplements were changed in accordance with the new additions to the text and the suggestions from the reviewers. Previous supplemental figures were changed to extended view figures in accordance to EMBO guidelines. Some figures were moved to the appendix.

Minor Points
Page 1, Line 27: "PFTs s" - extra s
Corrected
Page 2, Line 4: "mechanisms used PFTs" - missing by
Corrected
Page 3, Line 10: "this allowed to" - missing noun
Corrected
Page 3 Line 15: "the members of the hemolysin, the CPB..." - missing -like family
Corrected
Page 3 Line 20: "beta barrel protrusion" - rename to "top" or mention top from Xiong et al., 2022 Cell paper.
We consider top to be ambiguous. N-terminal beta-barrel protrusion is more precise.
Page 3, Line 26: "position 210" - Please label the residue in figure.
Figure has been corrected to include labeling of all relevant residues.
Page 3, Line 28: "tryptophane"- extra e
Corrected
Page 4, Line 25: "intracellular mouth" - intracellular is confusing, can a better name be used?
Changed to turns
Page 4, Line 30: "Supplementary Fig. 3D" - change to 3E
Corrected
Table 1: missing refinement statistics in table
Added the identifiers
Supplementary Table 1: "Sequence alignment Sequence alignment" - repeated twice. Citation for-matting in legend is not consistent (DOI). Please overlay CPB secondary structure on top of align-ment in the figure. Please add channel naming (rim, cap etc.)
Supplementary Figure 2: missing celsius. formatting for x g (check all instances throughout manu-script).
Corrected the figure legend and added the extra formatting.
Supplementary Figure 2: Please provide a micrograph of negatived-stained SMA oligomers.
Figure has been changed. Micrograph of cryo-em SMA sample has been added to a new supplementary figure.
Supplementary Figure 3: "putative rearrangements required...." - Missing sub panel F in figure?

Corrected
Supplementary Figure 3d: Please show a cross section of the resolution showing interior channel.
Added.
Supplementary Figure 4: Please report the color code in the legend. Black line in the graph is un-clear or missing.
Legend has been changed, figure is now EV3.
Supplementary Figure 4: "Nterminus" - Fix with N-terminus.
Fixed.
Supplementary Figure 7: Please add in legend or on graph, which the residues correspond to which constriction point in the channel.
New panel has been added to highlight the discussed residues and font has been changed for clarity.

Dear Dr. Zuber

Thank you for the submission of your revised manuscript to EMBO reports. It has been seen again by former referee #2, who supports publication without further revision.

Browsing through the manuscript myself, I noticed a few editorial things that we need before we can proceed with the official acceptance of your study.

- Your article will be published in our Reports section, which requires the merging of the Results and Discussion section.

- Please add a 'Disclosure and competing interests statement'. For more information see https://www.embopress.org/page/journal/14693178/authorguide#conflictsofinterest

- Appendix Fig S3 has panel A and B. Please add this information to the callout in line 33 (Appendix Fig S3A, B). The same applies to Appendix Fig S4 and its callout.

- Figure 2D legend: please define the nature of the 4 replicates (technical, biological/independent)

- Appendix table S1 is not really a table but rather a figure, and I therefore suggest to label it as Appendix figure S#.

- Appendix Fig. S4: Please define the scale bar in panel A.

- Please use bold upper case letters for the figure panels (e.g. A instead of a).
See also
https://www.embopress.org/pb-assets/embo-site/EMBOPress_Figure_Guidelines_061115-1561436025777.pdf

- Please note that we can only typeset up to 5 EV figures. Maybe you can merge two to reduce their number to 5?

- Data availability section: Please provide also a link that resolves to EMD-13876

- Please move the table to below the figure legends.

- Shall the map and model files be published as source data or as Dataset EV#? Please label them accordingly.

- Finally, EMBO reports papers are accompanied online by A) a short (1-2 sentences) summary of the findings and their significance, B) 2-3 bullet points highlighting key results and C) a synopsis image that is 550x200-600 pixels large (width x height) in .png format. You can either show a model or key data in the synopsis image. Please note that the size is rather small and that text needs to be readable at the final size. Please send us this information along with the revised manuscript.

We look forward to seeing a final version of your manuscript as soon as possible.

With kind regards,

Martina Rembold, PhD
Senior Editor
EMBO reports

****

Referee #2
[Is suitable for publication in EMBO reports without revision]

Dear Dr Rembold
Please find our final submission. Below you will find our answer to the tasks that you gave us.
Best wishes
Benoît Zuber et al.

- Your article will be published in our Reports section, which requires the merging of the Results and Discussion section.

We removed the Discussion section and re-fitted it in a Results and Discussion section.

- Please add a 'Disclosure and competing interests statement'. For more information see https://www.embopress.org/page/journal/14693178/authorguide#conflictsofinterest

We have added the section

- Appendix Fig S3 has panel A and B. Please add this information to the callout in line 33 (Appendix Fig S3A, B). The same applies to Appendix Fig S4 and its callout.

We have added the required info in the text (Fig S3 became Fig S4 and S4 became S5).

- Figure 2D legend: please define the nature of the 4 replicates (technical, biological/independent)

We have defined it in the figure legend.

- Appendix table S1 is not really a table but rather a figure, and I therefore suggest to label it as Appendix figure S#.

We have changed to it to Appendix Figure S1

- Appendix Fig. S4: Please define the scale bar in panel A.

A scale bar was added (100nm; now Appendix Fig. S5)

- Please use bold upper case letters for the figure panels (e.g. A instead of a).
See also
https://www.embopress.org/pb-assets/embo-site/EMBOPress_Figure_Guidelines_061115-1561436025777.pdf

All figures text and labels have been changed according to the embo guidelines.

- Please note that we can only typeset up to 5 EV figures. Maybe you can merge two to reduce their number to 5?

Two figures were merged to make a total of 5 EV Figures.

- Data availability section: Please provide also a link that resolves to EMD-13876

We cannot provide a link at this point. The EM map and the atomic model have been deposited on the PDB and the EMDB and are on hold. They will be released to the public upon publication. The PDB / EMDB codes are all that is needed to access the data.

- Please move the table to below the figure legends.

We have done it.

- Shall the map and model files be published as source data or as Dataset EV#? Please label them accordingly.

No this is not necessary, nor usual. The map and model files will be freely available on PDB and EMDB.

- Finally, EMBO reports papers are accompanied online by A) a short (1-2 sentences) summary of the findings and their significance, B) 2-3 bullet points highlighting key results and C) a synopsis image that is 550x200-600 pixels large (width x height) in .png format. You can either show a model or key data in the synopsis image. Please note that the size is rather small and that text needs to be readable at the final size. Please send us this information along with the revised manuscript.

We have added these items to our submission.

2nd Revision - Editorial Decision 14th Sep 2022

Benoît Zuber
University of Bern
Institute of Anatomy
Baltzerstrasse 2
Bern, BE 3012
Switzerland

Dear Dr. Zuber,

I am very pleased to accept your manuscript for publication in the next available issue of EMBO reports. Thank you for your contribution to our journal.

At the end of this email I include important information about how to proceed. Please ensure that you take the time to read the information and complete and return the necessary forms to allow us to publish your manuscript as quickly as possible.

As part of the EMBO publication's Transparent Editorial Process, EMBO reports publishes online a Review Process File to accompany accepted manuscripts. As you are aware, this File will be published in conjunction with your paper and will include the referee reports, your point-by-point response and all pertinent correspondence relating to the manuscript.

If you do NOT want this File to be published, please inform the editorial office within 2 days, if you have not done so already, otherwise the File will be published by default [contact: emboreports@embo.org]. If you do opt out, the Review Process File link will point to the following statement: "No Review Process File is available with this article, as the authors have chosen not to make the review process public in this case."

Thank you again for your contribution to EMBO reports and congratulations on a successful publication. Please consider us again in the future for your most exciting work.

Yours sincerely,

Martina Rembold, PhD
Senior Editor
EMBO reports

****

THINGS TO DO NOW:

Please note that you will be contacted by Wiley Author Services to complete licensing and payment information. The required 'Page Charges Authorization Form' is available here: https://www.embopress.org/pb-assets/embo-site/er_apc.pdf - please download and complete the form and return to embopressproduction@wiley.com

EMBO Press participates in many Publish and Read agreements that allow authors to publish Open Access with reduced/no publication charges. Check your eligibility: https://authorservices.wiley.com/author-resources/Journal-Authors/open-access/affiliation-policies-payments/index.html

You will receive proofs by e-mail approximately 2-3 weeks after all relevant files have been sent to our Production Office; you should return your corrections within 2 days of receiving the proofs.

Please inform us if there is likely to be any difficulty in reaching you at the above address at that time. Failure to meet our deadlines may result in a delay of publication, or publication without your corrections.

All further communications concerning your paper should quote reference number EMBOR-2022-54856V3 and be addressed to emboreports@wiley.com.

Should you be planning a Press Release on your article, please get in contact with emboreports@wiley.com as early as possible, in order to coordinate publication and release dates.

EMBO Press Author Checklist

Corresponding Author Name: Benoît Zuber	
Journal Submitted to: EMBO Reports	
Manuscript Number: EMBOR-2022-54856	

USEFUL LINKS FOR COMPLETING THIS FORM
The EMBO Journal - Author Guidelines
EMBO Reports - Author Guidelines
Molecular Systems Biology - Author Guidelines
EMBO Molecular Medicine - Author Guidelines

Reporting Checklist for Life Science Articles (updated January 2022)
This checklist is adapted from Materials Design Analysis Reporting (MDAR) Checklist for Authors. MDAR establishes a minimum set of requirements in transparent reporting in the life sciences (see Statement of Task: 10.31222/osf.io/9sm4x). Please follow the journal's guidelines in preparing your manuscript.
Please note that a copy of this checklist will be published alongside your article.

Abridged guidelines for figures
1. Data
The data shown in figures should satisfy the following conditions:
- the data were obtained and processed according to the field's best practice and are presented to reflect the results of the experiments in an accurate and unbiased manner.
- ideally, figure panels should include only measurements that are directly comparable to each other and obtained with the same assay.
- plots include clearly labeled error bars for independent experiments and sample sizes. Unless justified, error bars should not be shown for technical replicates.
- if n<5, the individual data points from each experiment should be plotted. Any statistical test employed should be justified.
- Source Data should be included to report the data underlying figures according to the guidelines set out in the authorship guidelines on Data Presentation.

2. Captions
Each figure caption should contain the following information, for each panel where they are relevant:
- a specification of the experimental system investigated (eg cell line, species name).
- the assay(s) and method(s) used to carry out the reported observations and measurements.
- an explicit mention of the biological and chemical entity(ies) that are being measured.
- an explicit mention of the biological and chemical entity(ies) that are altered/varied/perturbed in a controlled manner.
- the exact sample size (n) for each experimental group/condition, given as a number, not a range;
- a description of the sample collection allowing the reader to understand whether the samples represent technical or biological replicates (including how many animals, litters, cultures, etc.).
- a statement of how many times the experiment shown was independently replicated in the laboratory.
- definitions of statistical methods and measures:
  - common tests, such as t-test (please specify whether paired vs. unpaired), simple χ2 tests, Wilcoxon and Mann-Whitney tests, can be unambiguously identified by name only, but more complex techniques should be described in the methods section;
  - are tests one-sided or two-sided?
  - are there adjustments for multiple comparisons?
  - exact statistical test results, e.g., P values = x but not P values < x;
  - definition of 'center values' as median or average;
  - definition of error bars as s.d. or s.e.m.

Please complete ALL of the questions below.
Select "Not Applicable" only when the requested information is not relevant for your study.

Materials

Newly Created Materials	Information included in the manuscript?	In which section is the information available? (Reagents and Tools Table, Materials and Methods, Figures, Data Availability Section)
New materials and reagents need to be available; do any restrictions apply?	Not Applicable	

Antibodies	Information included in the manuscript?	In which section is the information available? (Reagents and Tools Table, Materials and Methods, Figures, Data Availability Section)
For **antibodies** provide the following information:<br>- Commercial antibodies: RRID (if possible) or supplier name, catalogue number and or/clone number<br>- Non-commercial: RRID or citation	Not Applicable	

DNA and RNA sequences	Information included in the manuscript?	In which section is the information available? (Reagents and Tools Table, Materials and Methods, Figures, Data Availability Section)
**Short novel DNA or RNA including primers, probes**: provide the sequences.	Yes	Material and Methods and Applendix Table S2

Cell materials	Information included in the manuscript?	In which section is the information available? (Reagents and Tools Table, Materials and Methods, Figures, Data Availability Section)
**Cell lines:** Provide species information, strain. Provide accession number in repository **OR** supplier name, catalog number, clone number, and/**OR** RRID.	Yes	Material and Methods
**Primary cultures:** Provide species, strain, sex of origin, genetic modification status.	Not Applicable	
Report if the cell lines were recently **authenticated** (e.g., by STR profiling) and tested for mycoplasma contamination.	Yes	

Experimental animals	Information included in the manuscript?	In which section is the information available? (Reagents and Tools Table, Materials and Methods, Figures, Data Availability Section)
**Laboratory animals or Model organisms:** Provide species, strain, sex, age, genetic modification status. Provide accession number in repository **OR** supplier name, catalog number, clone number, **OR** RRID.	Not Applicable	
**Animal observed in or captured from the field:** Provide species, sex, and age where possible.	Not Applicable	
Please detail **housing and husbandry conditions**.	Not Applicable	

Plants and microbes	Information included in the manuscript?	In which section is the information available? (Reagents and Tools Table, Materials and Methods, Figures, Data Availability Section)
**Plants:** provide species and strain, ecotype and cultivar where relevant, unique accession number if available, and source (including location for collected wild specimens).	Not Applicable	
**Microbes:** provide species and strain, unique accession number if available, and source.	Not Applicable	

Human research participants	Information included in the manuscript?	In which section is the information available? (Reagents and Tools Table, Materials and Methods, Figures, Data Availability Section)
If collected and within the bounds of privacy constraints report on age, sex and gender or ethnicity for all study participants.	Not Applicable	

Core facilities	Information included in the manuscript?	In which section is the information available? (Reagents and Tools Table, Materials and Methods, Figures, Data Availability Section)
If your work benefited from core facilities, was their service mentioned in the acknowledgments section?	Yes	Acknowledgements

Design

Study protocol	Information included in the manuscript?	In which section is the information available? (Reagents and Tools Table, Materials and Methods, Figures, Data Availability Section)
If study protocol has been **pre-registered, provide DOI in the manuscript**. For clinical trials, provide the trial registration number **OR** cite DOI.	Not Applicable	
Report the **clinical trial registration number** (at ClinicalTrials.gov or equivalent), where applicable.	Not Applicable	

Laboratory protocol	Information included in the manuscript?	In which section is the information available? (Reagents and Tools Table, Materials and Methods, Figures, Data Availability Section)
Provide DOI OR other citation details if **external detailed step-by-step protocols** are available.	Not Applicable	

Experimental study design and statistics	Information included in the manuscript?	In which section is the information available? (Reagents and Tools Table, Materials and Methods, Figures, Data Availability Section)
Include a statement about **sample size** estimate even if no statistical methods were used.	Yes	Table 1, Material and Methods, Appendix
Were any steps taken to minimize the effects of subjective bias when allocating animals/samples to treatment (e.g. **randomization procedure**)? If yes, have they been described?	Not Applicable	
Include a statement about **blinding** even if no blinding was done.	Not Applicable	
Describe **inclusion/exclusion criteria** if samples or animals were excluded from the analysis. Were the criteria pre-established?<br><br>If sample or data points were omitted from analysis, report if this was due to attrition or intentional exclusion and provide justification.	Not Applicable	
For every figure, are **statistical tests** justified as appropriate? Do the data meet the assumptions of the tests (e.g., normal distribution)? Describe any methods used to assess it. Is there an estimate of variation within each group of data? Is the variance similar between the groups that are being statistically compared?	Not Applicable	

Sample definition and in-laboratory replication	Information included in the manuscript?	In which section is the information available? (Reagents and Tools Table, Materials and Methods, Figures, Data Availability Section)
In the figure legends: state number of times the experiment was **replicated** in laboratory.	Yes	Figure 2 legend
In the figure legends: define whether data describe **technical or biological replicates**.	Yes	Figure 2 legend

Ethics

Ethics	Information included in the manuscript?	In which section is the information available? (Reagents and Tools Table, Materials and Methods, Figures, Data Availability Section)
Studies involving **human participants**: State details of **authority granting ethics approval** (IRB or equivalent committee(s), provide reference number for approval.	Not Applicable	
Studies involving **human participants**: Include a statement confirming that **informed consent** was obtained from all subjects and that the experiments conformed to the principles set out in the WMA Declaration of Helsinki and the Department of Health and Human Services Belmont Report.	Not Applicable	
Studies involving **human participants:** For publication of **patient photos**, include a statement confirming that consent to publish was obtained.	Not Applicable	
Studies involving experimental **animals**: State details of **authority granting ethics approva**l (IRB or equivalent committee(s), provide reference number for approval. Include a statement of compliance with ethical regulations.	Not Applicable	
Studies involving **specimen and field samples:** State if relevant **permits** obtained, provide details of authority approving study; if none were required, explain why.	Not Applicable	

Dual Use Research of Concern (DURC)	Information included in the manuscript?	In which section is the information available? (Reagents and Tools Table, Materials and Methods, Figures, Data Availability Section)
Could your study fall under dual use research restrictions? Please check biosecurity documents and list of **select agents and toxins** (CDC): https://www.selectagents.gov/sat/list.htm	Not Applicable	
If you used a select agent, is the security level of the lab appropriate and reported in the manuscript?	Not Applicable	
If a study is subject to dual use research of concern regulations, is the name of the **authority granting approval and reference number** for the regulatory approval provided in the manuscript?	Not Applicable	

Reporting

The MDAR framework recommends adoption of discipline-specific guidelines, established and endorsed through community initiatives. Journals have their own policy about requiring specific guidelines and recommendations to complement MDAR.

Adherence to community standards	Information included in the manuscript?	In which section is the information available? (Reagents and Tools Table, Materials and Methods, Figures, Data Availability Section)
State if relevant guidelines or checklists (e.g., **ICMJE, MIBBI, ARRIVE, PRISMA**) have been followed or provided.	Yes	Table 1
For **tumor marker prognostic studies**, we recommend that you follow the **REMARK** reporting guidelines (see link list at top right). See author guidelines, under 'Reporting Guidelines'. Please confirm you have followed these guidelines.	Not Applicable	
For **phase II and III randomized controlled trials**, please refer to the **CONSORT** flow diagram (see link list at top right) and submit the CONSORT checklist (see link list at top right) with your submission. See author guidelines, under 'Reporting Guidelines'. Please confirm you have submitted this list.	Not Applicable	

Data Availability

Data availability	Information included in the manuscript?	In which section is the information available? (Reagents and Tools Table, Materials and Methods, Figures, Data Availability Section)
Have **primary datasets** been deposited according to the journal's guidelines (see 'Data Deposition' section) and the respective accession numbers provided in the Data Availability Section?	Yes	Table 1, Material and Methods
Were **human clinical and genomic datasets** deposited in a public access-controlled repository in accordance to ethical obligations to the patients and to the applicable consent agreement?	Not Applicable	
Are **computational models** that are central and integral to a study available without restrictions in a machine-readable form? Were the relevant accession numbers or links provided?	Not Applicable	
If publicly available data were reused, provide the respective **data citations in the reference list**.	Not Applicable	
